# Landscape and dynamics of single tumor and immune cells in early and advanced‐stage lung adenocarcinoma

**DOI:** 10.1002/ctm2.350

**Published:** 2021-03-09

**Authors:** Zhencong Chen, Yiwei Huang, Zhengyang Hu, Mengnan Zhao, Ming Li, Guoshu Bi, Yuansheng Zheng, Jiaqi Liang, Tao Lu, Wei Jiang, Songtao Xu, Cheng Zhan, Junjie Xi, Qun Wang, Lijie Tan

**Affiliations:** ^1^ Department of Thoracic Surgery Zhongshan Hospital, Fudan University Shanghai China

**Keywords:** advanced/early lung adenocarcinoma, heterogeneity, single‐cell RNA‐seq, the tumor microenvironment

## Abstract

**Background:**

Lung adenocarcinoma (LUAD) patients with different American Joint Committee on Cancer stages have different overall 5‐year survival rates. The tumor microenvironment (TME) and intra‐tumor heterogeneity (ITH) have been shown to play a crucial role in the occurrence and development of tumors. However, the TME and ITH in different lesions of LUAD have not been extensively explored.

**Methods:**

We present a 204,157‐cell catalog of the TME transcriptome in 29 lung samples to systematically explore the TME and ITH in the different stages of LUAD. Traditional RNA sequencing data and complete clinical information were downloaded from publicly available databases.

**Results:**

Based on these high‐quality cells, we constructed a single‐cell network underlying cellular and molecular features of normal lung, early LUAD, and advanced LUAD cells. In contrast with early malignant cells, we noticed that advanced malignant cells had a remarkably more complex TME and higher ITH level. We also found that compared with other immune cells, more differences in CD8+/CTL T cells, regulatory T cells, and follicular B cells were evident between early and advanced LUAD. Additionally, cell‐cell communication analyses, revealed great diversity between different lesions of LUAD at the single‐cell level. Flow cytometry and qRT‐PCR were used to validate our results.

**Conclusion:**

Our results revealed the cellular diversity and molecular complexity of cell lineages in different stages of LUAD. We believe our research, which serves as a basic framework and valuable resource, can facilitate exploration of the pathogenesis of LUAD and identify novel therapeutic targets in the future.

## Graphical abstract

1. Our study comprehensively studied the tumor microenvironment (TME)and intra‐tumor heterogeneity (ITH) in different stages of LUAD by both scRNA‐seq and bulk RNA‐seq analyses.
2. Based on these high‐quality cells derived from different tissues, our results revealed the cellular diversity and molecular complexity of cell lineages in different stages of LUAD.
3. This study, which serves as a basic framework and valuable resource, can facilitate exploration of the pathogenesis of LUAD and identify novel therapeutic targets in the future.

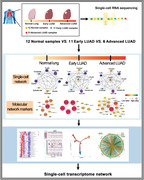

**Abbreviations**
DEGs: differential expression genes
GEO: gene expression omnibus
ITH: intra‐tumor heterogeneity
LUAD: lung adenocarcinoma
PPIs: protein‐protein interactions
RNA‐seq: RNA sequencing
scRNA‐seq: single‐cell sequencing
TCGA: The Cancer Genome Atlas
TME: tumor microenvironment
UMAP: uniform manifold approximation and projection
VEGF: vascular endothelial growth factor

## 1 INTRODUCTION

Lung cancer is the most common cancer, with more than 1,700,000 new cases every year.^1^, ^2^ The current histopathologic classification indicates that lung adenocarcinoma (LUAD) comprises the majority of all lung cancers. Although the treatment of LUAD has dramatically improved, LUAD patients with different American Joint Committee on Cancer stages exhibit different characteristics and survival,^3^, ^4^ indicating LUAD is more complicated than previously appreciated. Increasingly, the tumor microenvironment (TME), which plays a significant role in the occurrence and development of tumors, has been shown as a crucial source of intra‐tumor heterogeneity (ITH) in many studies.^5^, ^6^ ITH, which is recognized as a common characteristic of tumors, contributes to therapeutic failure, drug resistance, and ultimately lethal outcomes.^7^, ^8^

Consequently, it is essential to comprehensively characterize the TME and ITH in different stages of LUAD. Additionally, most studies investigating the transcriptome profile of LUAD were based on bulk RNA technologies, which can fail to detect the cellular diversity and molecular complexity of tumor cells.^9^ To compensate for the disadvantages of traditional RNA sequencing (RNA‐seq), 10x genomics single‐cell sequencing (scRNA‐seq), which is focused on cellular and molecular characteristics, has been widely used in tumor research.^10^, ^11^

Here, we comprehensively studied the TME and ITH in different stages of LUAD by both scRNA‐seq and bulk RNA‐seq analyses. Based on cells derived from different tissues, a single‐cell transcriptome atlas for non‐malignant lung tissues, early stage LUAD, and advanced stage LUAD was built. By analyzing the single‐cell transcriptome atlas, single‐cell networks for different lung conditions were constructed. We characterized the expression profiles of alveolar, early‐stage malignant, and advanced‐stage malignant cells at the single‐cell level. The cellular diversity and molecular complexity of immune cell lineages in different stages of LUAD were also explored. Moreover, our analysis established a landscape of cellular metabolism and communication for malignant and immune cells. In this study, we offer insight into the TME and ITH in the different stages of LUAD, which may help identify new therapeutic targets.

## 2 RESULTS

### 2.1 Single‐cell transcriptomic profiling from normal lung, early LUAD, and advanced LUAD tissues

**FIGURE 1:**
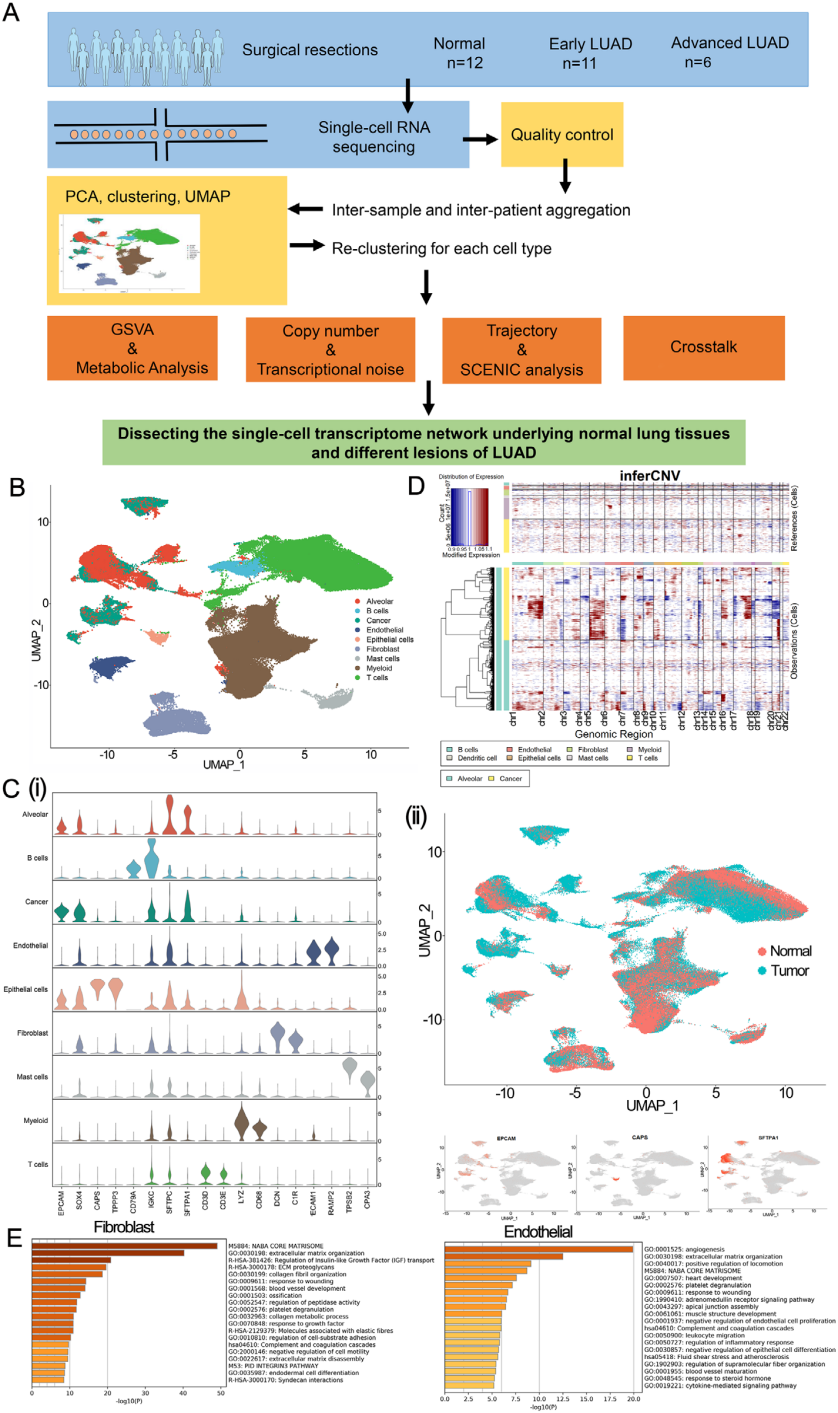
A single‐cell atlas of normal lung tissues, early LUAD, and advanced LUAD. (A) Workflow depicting collection and processing of specimens of normal lung, early LUAD, and advanced LUAD for scRNA‐seq analysis. (B) The UMAP plot and the proportion of 204,157 high‐quality cells to visualize cell‐type clusters based on the expression of known marker genes. (C) Expression of marker genes for each cell type. i. Violin plots displaying the expression of well‐known representative markers across each cell type identified in the non‐malignant and malignant lung tissues. The y‐axis shows the normalized read count. ii. The sample origin of the cells and feature plot of malignant/non‐malignant epithelial cells. (D) Heatmap showing large‐scale CNVs of each cell type. The expression values for cells (except alveolar and cancer cells) are plotted in the top heatmap, and the alveolar and cancer cells are plotted in the bottom heatmap, with genes ordered from left to right across the chromosomes. (E) The most enriched pathways for marker genes in fibroblast and endothelial cells, respectively

A total of 29 samples, including 12 normal lung samples (five from Human Cell Atlas Data database and seven from ArraryExpress database), 11 early LUAD samples (from Department of Thoracic Surgery, Zhongshan Hospital, Fudan University database [FDZSH]), and six advanced LUAD samples (three from FDZSH and three from ArraryExpress), were obtained from 26 patients in our study for downstream analysis (Figure 1A and Table 1). Among them, 13 were active/former smokers; the remaining were non‐smokers. The R package “Harmony” was applied to integrate the samples from different databases. As shown in Figure S1, cells grouped primarily by dataset were mixed after integration by the “Harmony” package, which showed the well‐integrated scRNA‐seq data. After quality control, a total of 204,157 cells that met the inclusion criteria were selected for subsequent analysis (including 82,065, 76,100, and 45,992 cells from normal samples, early LUAD, and advanced LUAD, respectively).

**TABLE 1:** Characteristics of the 26 LUAD patients included in this study for scRNA‐seq analysis

Sample	Age	Sex	Stage	Location	Gene mutation	Smoking history	Dataset
Patient 1	85‐90	Male	–	RUL	–	Former	ArrayExpress
Patient 2*	65‐70	Male	IIIB	RUL	–	Former	ArrayExpress
Patient 3*	60‐65	Female	IIB	LUL	–	Former	ArrayExpress
Patient 4	60‐65	Male	–	LUL	–	Former	ArrayExpress
Patient 5*	60‐65	Male	IIIA	LUL	–	Former	ArrayExpress
Patient 6	60‐65	Male	–	LUL	–	Former	ArrayExpress
Patient 7	50‐55	Female	–	RUL	–	Active	ArrayExpress
Patient 8	70‐75	Female	–	–	–	No	HCAD
Patient 9	40‐45	Female	–	–	–	No	HCAD
Patient 10	65‐70	Male	–	–	–	Former	HCAD
Patient 11	55‐60	Male	–	–	–	Active	HCAD
Patient 12	65‐70	Female	–	–	–	Non	HCAD
Patient 13	60‐65	Female	IB	RUL	EGFR	No	FDZSH
Patient 14	55‐60	Male	IB	LLL	EGFR	No	FDZSH
Patient 15	70‐75	Male	IB	LLL	EGFR	No	FDZSH
Patient 16	65‐70	Female	IB	RLL	EGFR	No	FDZSH
Patient 17	70‐75	Male	IB	LUL	EGFR	Active	FDZSH
Patient 18	75‐80	Male	IA	LUL	EGFR	No	FDZSH
Patient 19	60‐65	Female	IB	RUL	EGFR	Active	FDZSH
Patient 20	55‐60	Female	IB	RML	EGFR	Active	FDZSH
Patient 21	60‐65	Male	IA	LUL	EGFR	No	FDZSH
Patient 22	55‐60	Female	IA	RUL	HER‐2	No	FDZSH
Patient 23	50‐55	Female	IIB	LLL	EGFR	No	FDZSH
Patient 24	65‐70	Female	IB	RML	EGFR	No	FDZSH
Patient 25	70‐75	Male	IIA	RUL	EGFR	Active	FDZSH
Patient 26	45‐50	Female	IIIA	RUL	EGFR	No	FDZSH

**Abbreviations: FDZSH**, Department of Thoracic Surgery, Zhongshan Hospital, Fudan University; **HCAD**, human cell atlas data database; **LLL**, left lower lobe; **LUL**, left upper lobe; **RLL**, right lower lobe; **RML**, light middle lobe; **RUL**, right upper lobe.

*The patients provided the tumor and corresponding normal samples in our study.

We conducted dimensionality reduction and unsupervised clustering analysis to identify cell type and revealed nine cell type clusters. Then, based on the SingleR package, CellMarker dataset, and previous studies, we detected the following cell types: B cells (marked by CD79A and IGKC), T cells (marked by CD3D and CD3E), myeloid cells (marked by LYZ and CD68), fibroblasts (marked by DCN and C1R), endothelial cells (marked by PECAM1 and RAMP2), and mast cells (marked by TPSB2 and CPA3) (Figure 1B). To identify epithelial cells, cancer cells, and alveolar cells, we distinguished malignant and non‐malignant lung cells in the following ways. First, we used the SingleR package and the known markers of cancer and alveolar cells to identify a cancer cluster and alveolar cluster; EPCAM and SOX4 were used to mark the cancer cluster; SFTPC and SFTPA1 to mark the alveolar cluster; CAPS and TPPP3 to mark epithelial cells. Detailed information of the marker genes for each cluster is shown in Figure 1C and Figure S2A. Subsequently, we identified cells derived from LUAD samples, and those with high expression levels of tumor marker genes were identified as "cancer cluster." Based on cellular origin, we found that "cancer cluster" cells were all derived from LUAD samples. By contrast, cells in the "alveolar cluster” and "epithelial cluster” were mostly derived from normal lung tissues (Figure 1C), strongly indicating that cells in "cancer cluster" should be malignant cells and cells in “alveolar cluster” and “epithelial cluster are normal lung cells.

Previous studies have shown that compared with other normal cells, malignant cells had significantly higher copy number variations (CNVs); thus, the "infercnv" package was applied to calculate CNVs for “Cancer cluster” and “Alveolar cluster,” respectively (Supplementary Materials Details). In Figure 1D, reference cells defined as normal cells are plotted in the top heatmap, while cells in the “alveolar cluster” and “Cancer cluster” are plotted in the bottom heatmap. Compared with reference cells, we observed that the “cancer cluster” had remarkably higher CNV levels than reference cells, while the “alveolar cluster” had similar CNV levels with reference cells. Moreover, we also re‐clustered the alveolar and cancer cells and plotted these cells with each cell color‐coded for its sample type of origin, the associated cell type, and malignancy score (which was calculated based on the CNV value). As shown in Figure S2B, cancer cells were all derived from LUAD samples, while alveolar cells originated from both LUAD and non‐malignant samples. We also noticed that cancer cells had a higher malignancy score than alveolar cells. Overall, these studies suggested reasonable cell annotation in our study (Figure 1B).

The “scPred” package was also applied to validate our results (Supplementary Materials Details). As shown in Figure S3, we noticed that most cells that were identified as “cancer cluster” cells in Seurat object were not classified as “alveolar cluster” or “epithelial cluster” cells in scPred object and cells identified as “Alveolar cluster” or “epithelial cluster” in the Seurat object were also identified as non‐malignant lung cells in the scPred object. Thus, our scPred results confirmed the identification of cancer and non‐malignant cells.

To characterize each cell population, functional enrichment analyses based on cell type gene markers were performed for each cell population in our study (Supplementary Materials Details). For example, as shown in Figure 1E, fibroblast cells are mainly involved in extracellular matrix organization, and endothelial cells play an essential role in angiogenesis. Detailed information of the functional enrichment analyses for other cell types is shown in Figure S4.

### 2.2 Construction of the single‐cell network in normal lung tissues and different lesions of LUAD

**FIGURE 2:**
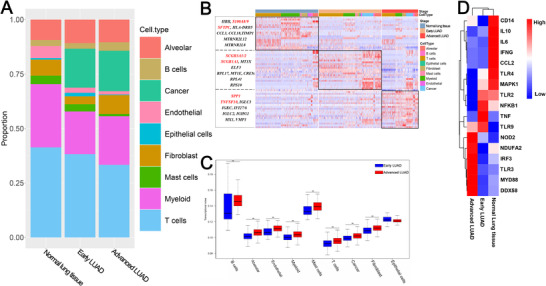
The single‐cell transcriptomes of each cell type in normal lung tissues and different conditions of LUAD. (A) The proportion of each cell type in normal lung, early LUAD, and advanced LUAD. (B) Heatmap showing the expression patterns of multiple cell types in normal lung tissues and different conditions of LUAD (only shown top 10 DEGs in each condition of lung samples). (C) Boxplot illustrates transcriptional noise by conditions of LUAD and cell type for the indicated number of cells. For all boxplots, the box represents the interquartile range, the horizontal line in the box is the median, and the whiskers represent 1.5 times the interquartile range. Blue and red colors indicate early stage and advanced stage cells, respectively. The asterisk indicates significant changes (Wilcoxon's rank‐sum test, adjusted *p*‐value < 0.05). Cell types are ordered by decreasing the transcriptional noise ratio between advanced and early cells. (D) The expressions of innate immune response‐associated genes across the group

To characterize the single‐cell transcriptome from normal lung tissues to advanced LUAD, 204,157 cells were selected for subsequent analysis. As shown in Figure 2A, the proportion of myeloid cells increased significantly in non‐malignant lung samples, fibroblast cells increased significantly in advanced LUAD cells, and T cells had similar proportions among different types of lung samples.

Next, the Wilcoxon rank‐sum test was used to detect differentially expressed genes (DEGs) in different lung sample conditions (false discovery rate [FDR] < 0.01 and fold change > 0.5). Then, the DEGs in the same lung sample conditions were merged and set as condition‐related genes (Figure 2B and Table S1). As shown in Figure 2B, we found that compared with other conditions, genes related to anti‐inflammatory and poor prognosis (e.g., SPP1 and TNFSF10) were mainly detected in advanced LUAD conditions,^12^, ^13^ while genes related to inflammation and normal alveolar function (e.g., SFTPC and S100A8) were mainly identified in normal lung samples.^14^, ^15^ Additionally, we also found that SCGB3A1/2 and SCGB1A1 were enriched in early LUAD. Previous studies had reported that these genes belong to the cytokine‐like small molecular weight secreted protein family and play a vital anti‐inflammatory role in many tumors, especially lung tumors.^16^, ^17^ Overall, our research revealed unique condition‐related genes in each lung sample condition. Functional enrichment analyses revealed that early LUAD condition‐related genes were mainly enriched in metabolic pathways, while condition‐related genes for advanced LUAD were closely related to cell proliferation and differentiation (Figure S5).

It is known that transcriptional noise plays an essential role in heterogeneity.^18^ Thus, we determined the transcriptional noise for each cell type by exploring cell‐cell variance shifts in different LUAD lesions (Supplementary Materials Details). As shown in Figure 2C, in contrast with cells in early LUAD, most cells (except epithelial cells) in advanced LUAD had higher transcriptional noise, especially B cells and alveolar cells. Previous studies have demonstrated that the primary function of B cells is the innate immune response.^19^, ^20^ Therefore, we explored innate immune response‐associated genes^21^ across the groups.

We observed that genes that play an essential role in promoting inflammation (e.g., IL6, IL10, and CCL2) were highly expressed in normal lung samples (Figure 2D). Intriguingly, we also noticed that some innate immune response‐related genes (e.g., TLR3, MYD88, and DDX5) were mainly enriched in the advanced LUAD group. Some have been well characterized in LUAD. For example, TLR3, which belongs to the family of type I integral membrane‐associated receptors, has been reported to play an important role in immune and inflammatory responses.^22^ Tavora et al^23^ reported that TLR3 could induce the expression of SLIT2 and promote cancer progression. Further investigation of the expression of innate immune response‐related genes B cells will be important.

**FIGURE 3:**
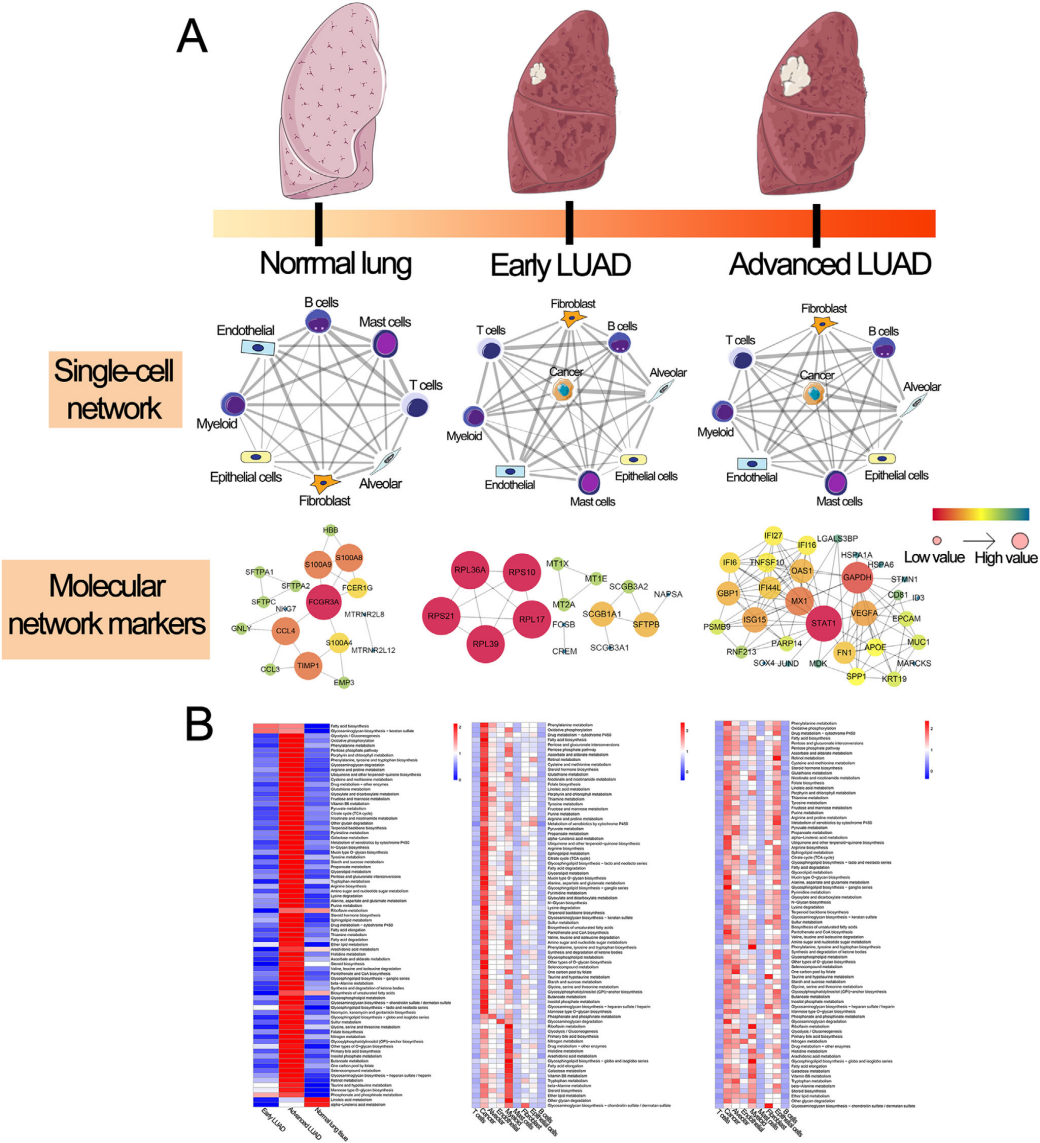
The single‐cell transcriptome network underlying non‐malignant lung, early LUAD, and advanced LUAD. (A) Cellular and molecular changes from normal lung tissues to advanced LUAD. Top: The nodes stand for each epithelial cell type in ESCC and non‐malignant esophageal tissue, and the thickness of edges in the network denotes the Pearson correlation coefficient between each cell type. Bottom: Protein‐protein interactions (PPIs), which were based on the signatures (FDR < 0.01 and fold change > 1.5) for each representative cell type in different stages. The size and color for nodes represent the combined scores of PPIs. (B) Metabolic pathway activities in different lung samples. Left: Metabolic pathway activities in normal lung, early LUAD, and advanced LUAD. Centre: Metabolic pathway activities in early LUAD. Right: Metabolic pathway activities in advanced LUAD

To comprehensively characterize both cellular and molecular changes from normal lung tissues to advanced LUAD, a single‐cell transcriptomic network was built by describing the correlations between each pair in the cell populations (followed by detection of marker genes for each cell type) in normal lung tissues and different lesions of LUAD (Figure 3A). Protein‐protein interaction (PPI) analysis for primary cell types and metabolism analysis for each cell type was also performed to show dynamic alterations in each lung sample condition. We observed a strong connection between each cell population in normal lung tissues, early LUAD, and advanced LUAD (Figure 3A).

In metabolic re‐programming analysis, we noticed that in contrast with other cell types, the upregulated metabolic pathways were mainly enriched in cancer cells and myeloid cells in early LUAD. By contrast, for advanced LUAD, the upregulated metabolic pathways mostly appeared in cancer, alveolar, and epithelial cells, which revealed the different metabolic re‐programming patterns in different lesions of LUAD (Figure 3B and Table S2). These results revealed that the metabolic reprogramming of cell type within the TME (especially glycolytic and oxidative phosphorylation pathways) might block the anti‐tumor effects of immune cells; this has not been previously reported in LUAD at the single‐cell level.

### 2.3 Malignant cell differences across lesions and tumor clonal heterogeneity

**FIGURE 4:**
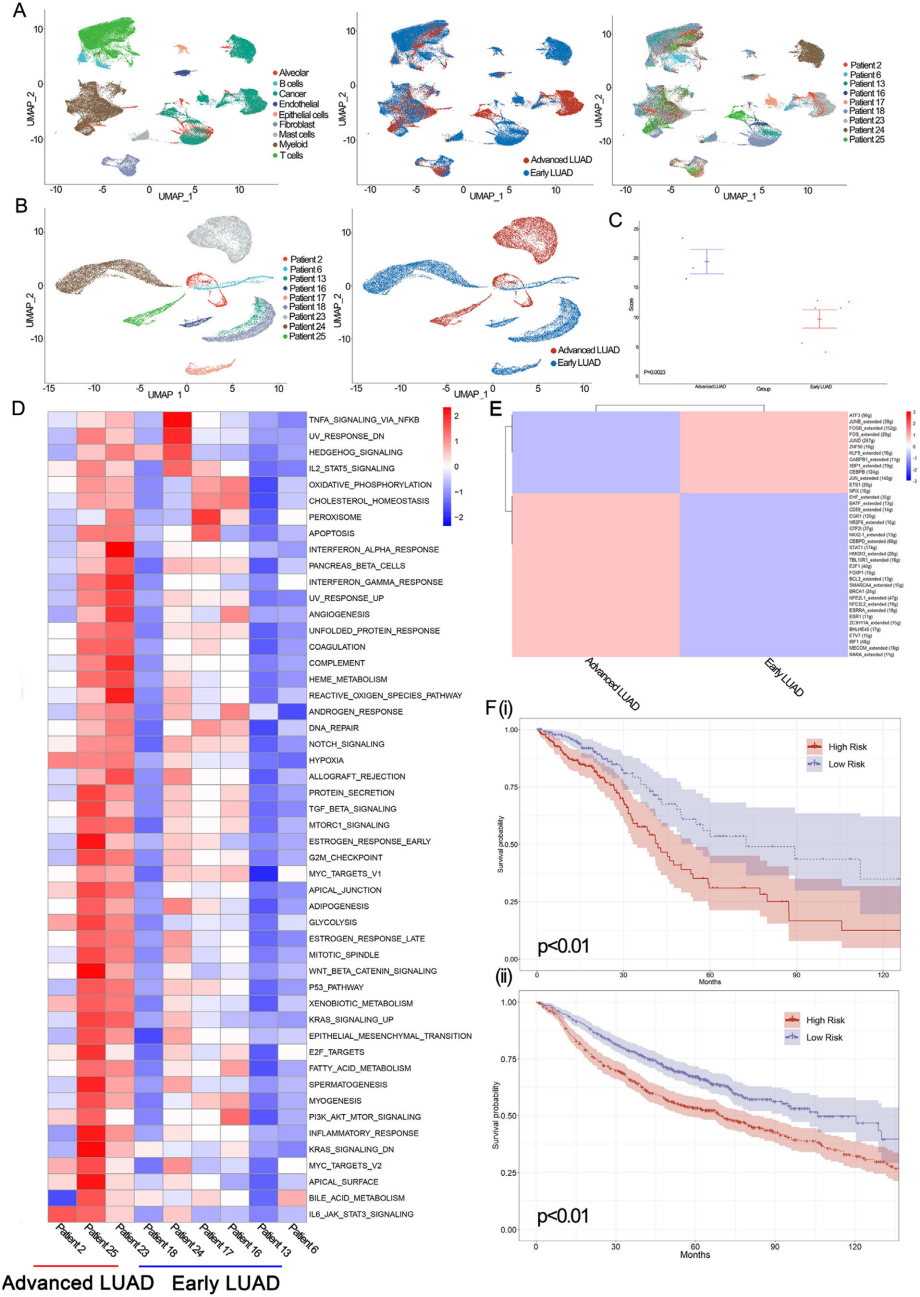
The scRNA and bulk profiles for malignant cell lineages in early LUAD and advanced LUAD. (A) UMAP plot of 68,089 tumor‐derived cells from nine LUAD patients, with each cell color‐coded for (left to right): the associated cell type, its sample type of origin (early or advanced LUAD), and the corresponding patient. (B) UMAP plot of 21,861 LUAD cells from nine LUAD patients, with each cell color‐coded for (left to right): the corresponding patient and its sample type of origin (early LUAD or advance LUAD). (C) Diversity score of LUAD samples. Data are presented as means ± SEM. (D) Pathway activities were scored in nine LUAD patients using GSVA. (E) SCENIC analysis for LUAD cells from early LUAD or advance LUAD. (F) Kaplan‐Meier (KM) survival curve of five advanced LUAD‐related prognostic signatures. i. KM‐ survival curve in the TCGA database. ii. KM‐ survival curve in the GEO database

We selected patients with more than 500 cancer cells for downstream analysis; nine patients with 68,089 tumor‐derived cells (six early LUAD and three advanced LUAD) were included (Figure 4A). Heterogeneity, which is well known as a prominent characteristic of tumors, is considered an important cause of therapeutic failure, drug resistance, and ultimately lethal outcomes. Thus, it makes sense to investigate heterogeneity in LUAD, especially in malignant cells. As shown in Figure 4B, we observed that 21,861 malignant cells formed patient‐specific clusters, which revealed significant heterogeneity in each patient. To further explore whether the level of ITH in advanced LUAD was higher than in early LUAD, we applied a formula to estimate tumor cell‐specific transcriptomic diversity scores in our study (Supplementary Materials Details). Intriguingly, the diversity scores in advanced LUAD were higher than in early LUAD (*p* = 0.023); in particular, the top three diversity scores were all from advanced LUAD (Figure 4C).

Next, to evaluate the role of heterogeneity in biological function, we performed GSVA analysis of malignant cells from each patient (Supplementary Materials Details). As shown in Figure 4D, our results suggested that compared with early malignant cells, pathways were mostly upregulated in advanced malignant cells, especially in patient 25. Although mainly pathways had higher expression levels in advanced tumor cells, pathways involved KRAS_SIGNALING_DN, WNT_BETA_CATENIN_SIGNALING, and ESTROGEN_RESPONSE_EARLY were preferentially expressed in patient 25, while pathways involved REACTIVE_OXIGEN_SPECIES_PATHWAY, INTERFERON_ALPHA_RESPONSE, and ANGIOGENESIS were expressed in patient 23. Moreover, we also found that pathways associated with reactive oxygen species (ROS), inflammation, and angiogenesis were upregulated in advanced LUAD patients (Figure 4D). Previous studies have suggested that pathways associated with ROS and apoptotic pathways can contribute to inflammation^24^, ^25^, ^26^; therefore, we evaluated ROS and apoptotic gene expression signatures between advanced and early LUAD groups (Supplementary Materials Details). Then, as shown in Figure S6A and Table S3, advanced LUAD patients had significantly higher expression levels of MPO, FEZ1, PPP2R4, and CREBBP than early LUAD. We also performed qRT‐PCR to confirm our results (Figure S6B and S6C). In summary, our study confirmed both inter‐ and intra‐tumoral transcriptomic heterogeneity in different LUAD lesions.

To better describe the gene regulatory network between early and advanced tumor cells, we conducted SCENIC analysis (Supplementary Materials Details). Genes regulated by EHF_extended, BATF_extended, and CD59_extended were highly upregulated in advanced tumor cells, while genes regulated by ATF3, JUNB_extended, and FOSB_extended were upregulated in early tumor cells (Figure 4E). Next, to characterize the single‐cell expression profiles for malignant cells in different lesions of LUAD, differential expression analysis was applied in our study. As a result, we found that TNFSF10, ECM1, and RNF213 were the top three upregulated genes in advanced LUAD. By contrast, early LUAD had increased expression of SCGB3A2, SCGB3A1, and SFTPC (Table S4). To validate the marker genes of different conditions of LUAD in our study, flow cytometry and qRT‐PCR were performed. As shown in Figure S6 based on EPCAM, a marker of malignant cells, tumor cells in early and advanced LUAD were selected. Our qRT‐PCR results revealed that the expression levels of TNFSF10 (*p* < 0.01), ECM1 (*p* < 0.01), and RNF213 (*p* < 0.01) were significantly increased in advanced LUAD tumor cells, whereas the expression of SCGB3A2 (*p* < 0.01), SCGB3A1 (*p* < 0.01), and SFTPC (*p* < 0.01) was increased in early LUAD (Figure S6B and S6C). These findings indicated that these genes could serve as new alarm signals for LUAD.

Furthermore, based on gene markers for advanced LUAD, 454 LUAD patients in The Cancer Genome Atlas (TCGA) database and 1061 LUAD patients in the gene expression omnibus (GEO) database were selected to identify prognostic markers for LUAD patients (Supplementary Materials Details). Consequently, CEACAM6, CTSE, SQSTM1, and VEGFA were identified as prognostic stage‐related genes in our study (Figure S7). As shown in Figure 4F, we found that in the prognostic model, which was contrasted by stage‐related genes, patients were divided into high‐risk and low‐risk groups in both the TCGA and GEO databases, suggesting that our prognostic model can accurately predict the prognosis of LUAD patients.

### 2.4 Characterization of the single‐cell expression profiles for immune cells and lineages in different lesions of LUAD

**FIGURE 5:**
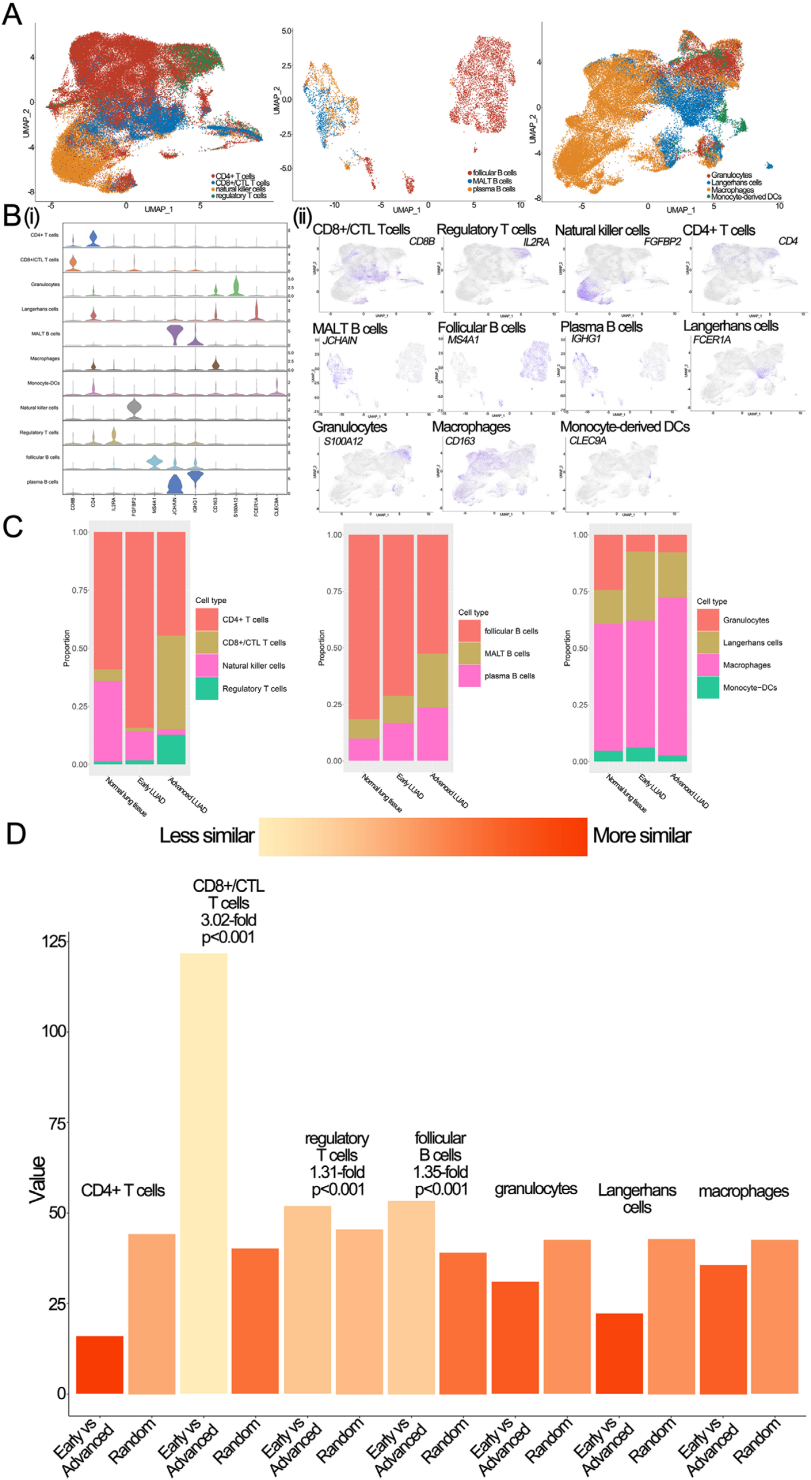
The scRNA profiles for immune cell lineages in normal lung, early LUAD, and advanced LUAD. (A) UMAP plot of T cells, B cells, and Myeloid cells, respectively. (B) Violin plots and feature plots of immune marker genes. i. Violin plots. ii. Feature plots. (C) The proportion of T cells, B cells, and myeloid cells, respectively. (D) Quantification of differences between major immune lineages in early LUAD and advanced LUAD. Each dot stands for a sub‐sample of 400 cells from PCA space for early LUAD and advanced LUAD or a sample of 400 cells from a random group. The height of the bar is the mean of the subsample

Immune cells are an essential part of the TME and play an important role in tumor development and therapy failure. Thus, it is necessary to compare the immune cells and lineages in different lesions of LUAD for the development of novel therapeutic targets. Here, to comprehensively investigate immune cells, we re‐clustered T cells, B cells, and myeloid cells (Figure 5A). T cells were divided into CD8+/CTL T cells (marked by CD8B), CD4+ T cells (marked by CD4), regulatory T cells (marked by IL2RA), and natural killer cells (marked by FGFBP2); B cells were re‐clustered as follicular B cells (marked by MS4A1), MALT B cells (marked by JCHAIN), and plasma B cells (marked by IGHG1); myeloid cells were divided into macrophages (marked by CD163), granulocytes (marked by S100A12), Langerhans cells (marked by FCER1A), and monocyte‐derived dendritic cells (marked by CLEC9A and DUSP4). Detailed information of the marker genes for each cluster is shown in Figure 5B.

To investigate the similarities of these immune lineages between early and advanced LUAD, we calculated the proportions of each cell proportion for each LUAD lesion in our study. We found that compared cell proportions within early LUAD to those within advanced LUAD, we observed the frequencies of CD8+/CTL T cells, regulatory T cells, and macrophages were significant increases in advanced LUAD. By contrast, the frequency of follicular B cells, Langerhans cells, and CD4+ T cells decreased significantly in advanced LUAD (Figure 5C). Next, we assessed CD8+/CTL T cells, regulatory T cells, macrophages, follicular B cells, Langerhans cells, and CD4+ T cells to study the changing immune trend in HLA‐related genes. As shown in Figure S8, we found that major histocompatibility complex (MHC) class I molecules (e.g., HLA‐A, HLA‐B, and HLA−C) were highly expressed on all immune cells. By contrast, MHC II molecules (e. g., HLA‐DQA1/2, HLA‐DMA/B, and HLA−DRA/B1) were only overexpressed in cells with decreased frequency in early LUAD, especially in macrophages. Previous studies have demonstrated that MHC II can mediate the presentation process of exogenous antigens.^27^ Alspach and colleagues reported that activation of CD4+ T cells by MHC II molecules could play an important anti‐tumor role.^28^ Our study showed that compared to macrophages in early LUAD, macrophages in advanced LUAD had significantly lower MHC II gene expression. Together, our results indicate that the elevation of macrophages with lower expression of MHC II molecules and the decrease in CD4+ T cells in advanced LUAD might explain the immune escape of LUAD, and MHC II overexpressing macrophages could be a potential immune therapy target.

**FIGURE 6:**
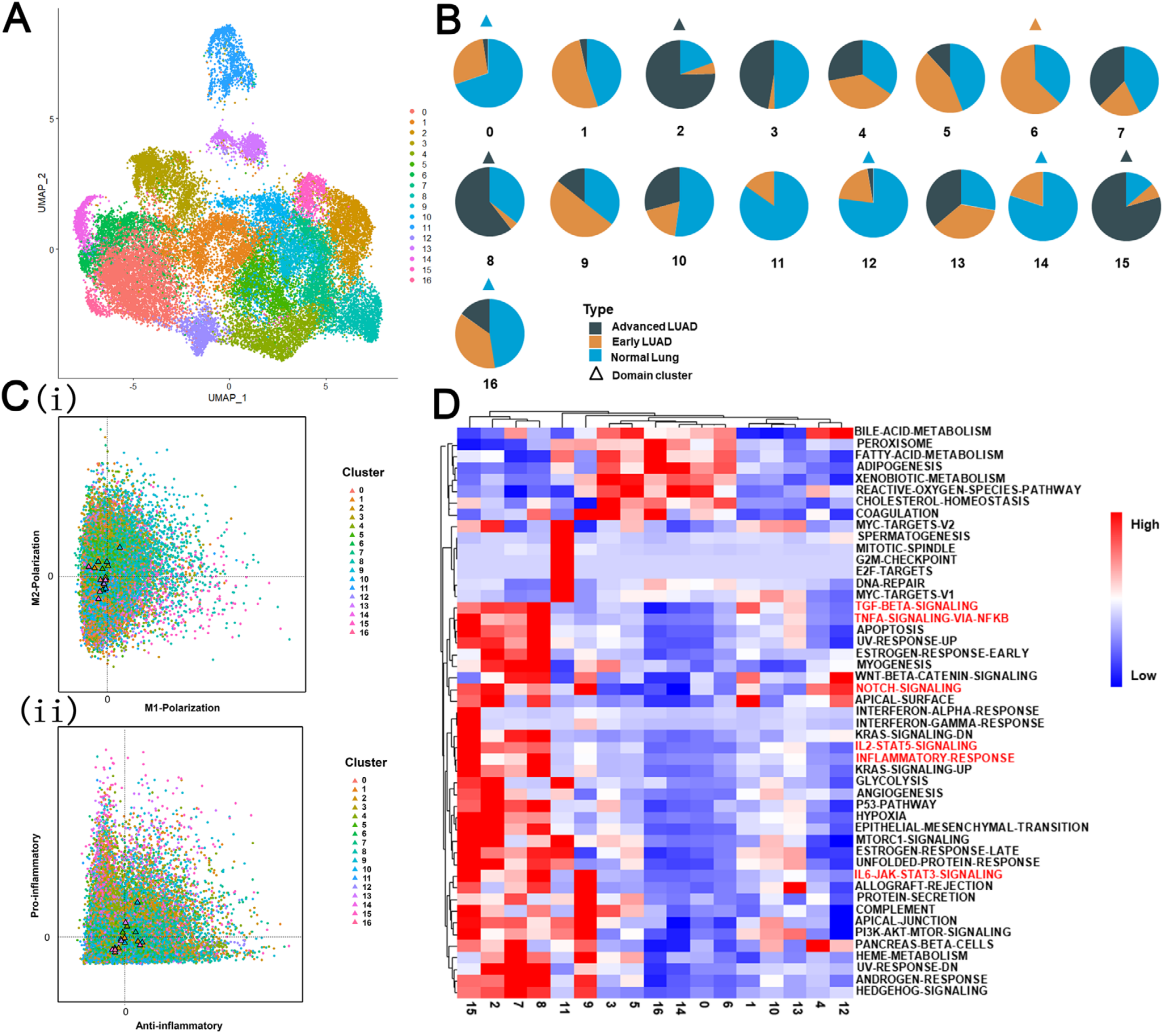
The scRNA profiles for macrophages in normal lung, early LUAD, and advanced LUAD. (A) UMAP plot of macrophages in normal lung, early LUAD, and advanced LUAD. (B) Pie charts showing the proportions of cells originating from each condition of lung samples detected in each cluster, colored by condition. (C) Scatterplots showing M1 and M2 scores (left panel) and pro‐ and anti‐inflammatory scores (right panel) for each color‐coded macrophage clusters. (D) Pathway activities scored in all 17 macrophage clusters

We then focused on myeloid cells and found that although myeloid cells were present in a higher proportion in non‐malignant patients than early and advanced LUAD patients, macrophages cell represented the most prevalent cell type in myeloid cells at different conditions of lung samples (Figure 5C). We also noticed that the proportions of macrophages gradually increased with disease progression. Together, these results suggest that macrophages play an essential role in the development of LUAD. Therefore, we re‐clustered macrophages and identified a total of 17 sub‐clusters (Figure 6A). We analyzed cellular contributions to each cluster and found that eight clusters had cells (≥60%) from the sole individual state (Figure 6B); we defined these eight clusters as "domain" clusters (including four normal lung clusters, one early LUAD domain cluster, and three advanced LUAD domain clusters). These results indicate a pervasive heterogeneity in macrophages.

Next, to study the relationship between macrophages and disease progression, we explored macrophage function. We calculated the M1 and M2 polarization and pro‐inflammatory and anti‐inflammatory scores based on previous studies.^29^ As shown in Figure 6C, both M2‐like and anti‐inflammatory phenotypes in advanced LUAD domain clusters (clusters 2, 8, and 15) were identified. Next, we performed GSVA analysis among all 17 sub‐clusters to further evaluate functional changes in different clusters. As shown in Figure 6D, we found that inflammatory pathways (e.g., IL6/STAT3, Notch, and IL2‐STAT5 signaling pathways) related to poor prognosis were mainly enriched in advanced LUAD domain clusters. Overall, our results revealed that macrophages in advanced LUAD have a dominant anti‐inflammatory phenotype, which could promote LUAD growth.

The distance between each type of immune cell (including 400 cells in each group) was also estimated by Bhattacharyya distance (Supplementary Materials Details). Obvious differences between early and advanced LUAD were identified in CD8+/CTL T cells (3.02‐fold change, *p* < 0.001), follicular B cells (1.35‐fold change, *p *< 0.001), and regulatory T cells (1.31‐fold change, *p* < 0.001); while other cell types, such as myeloid cells, were less similar between early and advanced LUAD (Figure 5D). Therefore, we selected CD8+/CTL T, regulatory T, and follicular B cells for downstream analysis.

**FIGURE 7:**
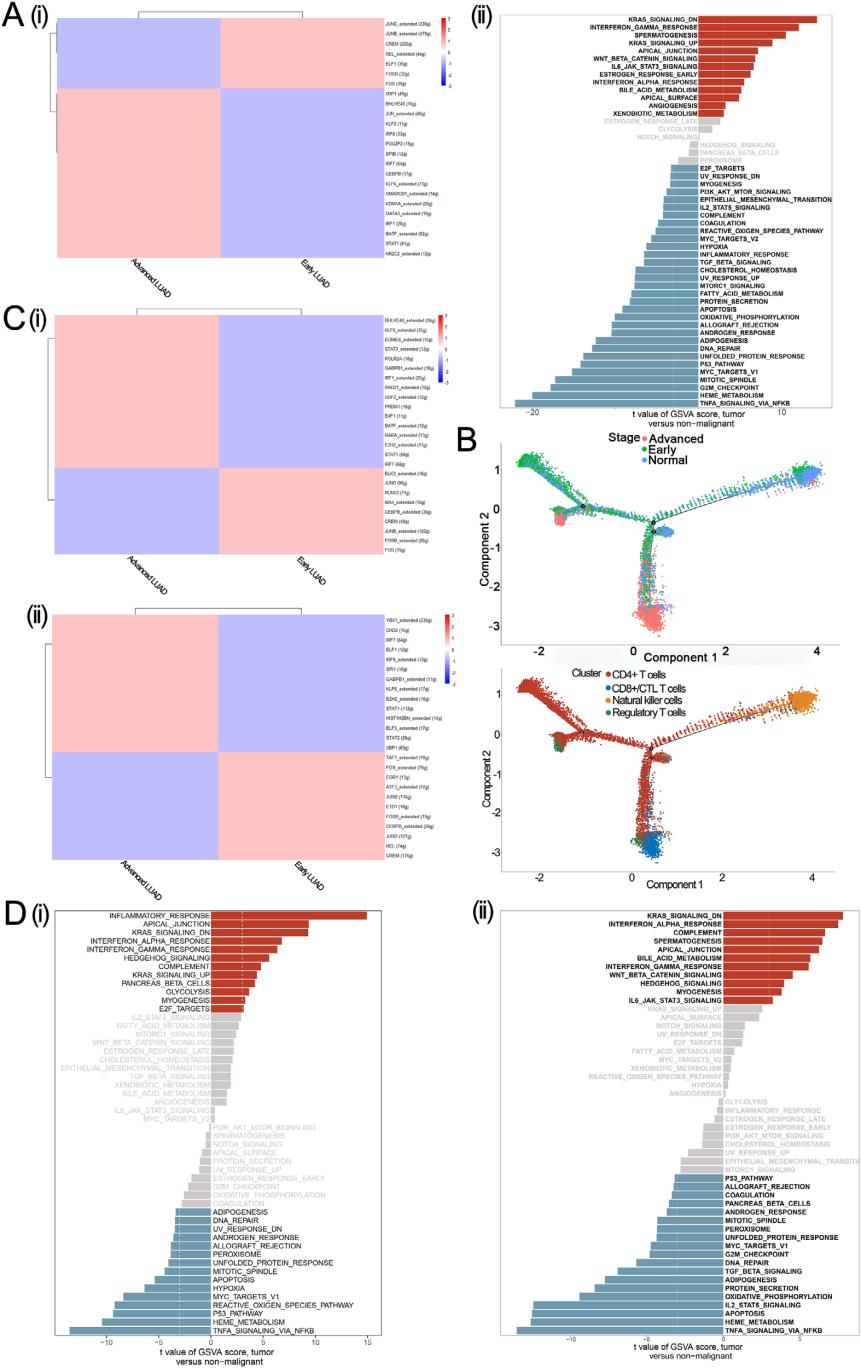
Trajectory analysis, and gene set variation analysis, and SCENIC analysis for T Cells, B cells, and myeloid cells. (A) i. SCENIC analysis for follicular B cells. ii. GSVA analysis for follicular B cells. (B) Trajectory analysis in T cells. (C) SCENIC analysis for T cells. i. SCENIC analysis for CD8+/CTL T cells. ii. SCENIC analysis for regulatory T cells. (D) GSVA analysis for T Cells. i. GSVA analysis for CD8+/CTL T cells. ii. GSVA analysis for regulatory T cells

We selected 2345 follicular B cells (954 from the early LUAD group and 1391 from the advanced LUAD group) for subsequent analysis. Differential expression analysis revealed that HLA‐DRB5, PSMB9 IGLC3, and PSMB9 were mostly expressed in advanced follicular B cells, whereas MT2A and RPL41 were mainly detected in early follicular B cells (Table S4). Gene regulatory analysis showed that genes regulated by JUND_extended, JUNB_extended, and CREM were mainly enriched in early follicular B cells, while genes regulated by XBP1, BHLHE40, and JUN_extended were highly upregulated in advanced follicular B cells (Figure 7A). We also found that advanced follicular B cells were enriched in INTERFERON_GAMMA_RESPONSE, KRAS_SIGNALING_DN, and SPERMATOGENESIS compared to early follicular B cells (Figure 7A).

To quantitatively track T cell reprogramming in the normal, early LUAD, and advanced LUAD state, we performed trajectory analysis (Supplementary Materials Details). As shown in Figure 7B, the state that only contains a single cell type (cells in this state were primarily from normal tissue) was set as the “root” state in our study. Our results showed differentiation paths from normal‐specific natural killer cells to tumor‐derived CD8+/CTL T cells, tumor‐derived CD4+ T cells, and tumor‐derived regulatory T cells. Focusing on the branch from root to early and advanced LUAD, we found that CD4+ T cells were observed as an intermediate state, while CD8+/CTL and regulatory T cells were mainly exited in the advanced LUAD group. These findings show the complex reprogramming of T cells between early and advanced LUAD.

Next, we focused on CD8+/CTL and regulatory T cells. A total of 6597 CD8+/CTL T cells (including 417 early LUAD and 6150 advanced LUAD group cells) were evaluated in our study. Differential expression analysis was applied to identify distinct signatures in early or advanced CD8+/CTL T cells. We noticed that advanced CD8+/CTL T cells had higher expression of CXCL13, HAVCR2, and RBPJ, while RPL41, EEF1A1, and RPL39 were the top three upregulated genes in early CD8+/CTL T cells (Table S4). For regulatory T cells, a total of 2396 cells (with 474 early LUAD and 1922 advanced LUAD group cells) were obtained for differential expression analysis. Advanced regulatory T cells had higher expression of CXCL13, IFI27, and CCL5, while LMNA, MT2A, and RPL39 were overexpressed in early regulatory T cells (Table S4). These results indicated that compared to early LUAD, a higher infiltration of CD8 cytotoxic and regulatory T cells expressing CXCL13 and a lower infiltration of follicular B cells in advanced LUAD could constitute a prognostic factor for a patient with LUAD, consistent with previous studies.^30^, ^31^ SCENIC analysis revealed that advanced CD8+/CTL T cells had a greater activity of BHLHE40_extended and KLF6_extended, whereas advanced regulatory T cells had higher activity of YBX1_extended and CHD2 (Figure 7C). We also found that advanced CD8+/CTL T cells were more closely related to INFLAMMATORY_RESPONSE, APICAL_JUNCTION, and KRAS_SIGNALING_DN pathways, while KRAS_SIGNALING_DN, INTERFERON_ALPHA_RESPONSE, and COMPLEMENT pathways were over‐upregulated in advanced regulatory T cells (Figure 7D). In summary, our results suggested that enrichment for activation of KRAS signaling in advanced LUAD could be a potential therapeutic target.

### 2.5 Crosstalk between cancer and immune cells

Tumors are heterogeneous mixtures of cells, and crosstalk between cancer and immune cells has been shown to play an important role in the evolution of tumors. The R package “CellTalker” was used to evaluate the interactions between cancer and immune cells. We classified cell‐cell communications into common interactions (interactions existing in more than two lung sample conditions) and unique interactions (interactions only occurring in one lung sample condition).

**FIGURE 8:**
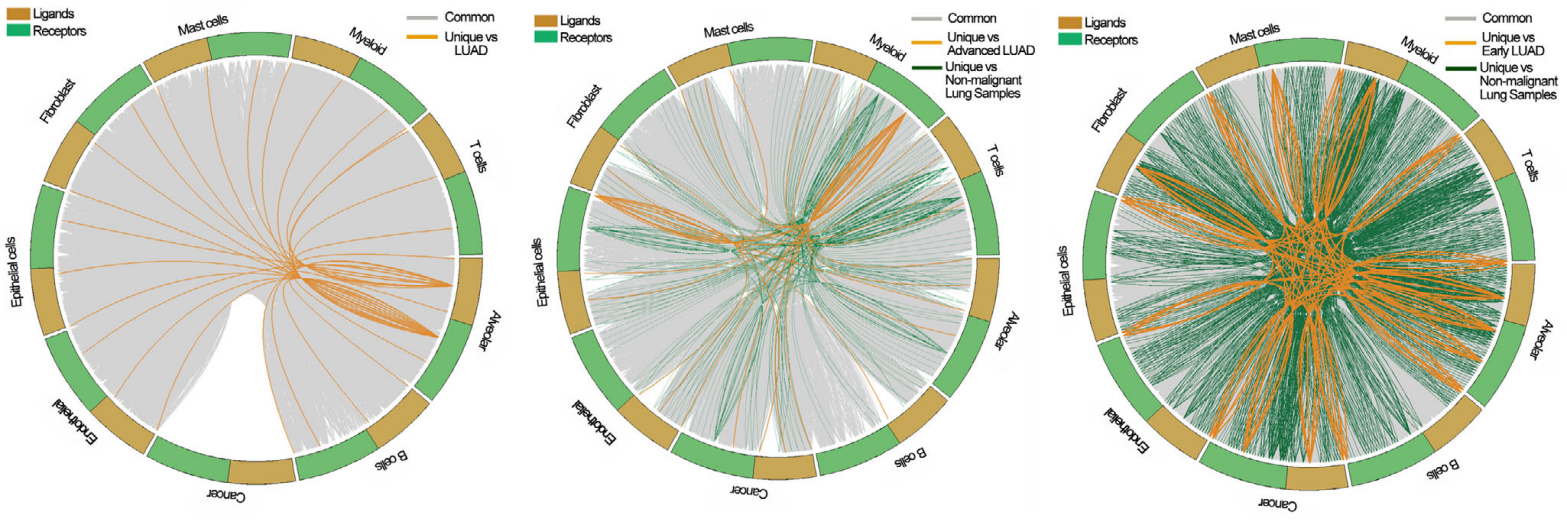
Circos plot showing the interactions between ligands and receptors across cell types in non‐malignant lung, early LUAD, and advanced LUAD. Left to right: the cell‐cell communications in between normal lung and LUAD, the cell‐cell communications in between early LUAD and other lung samples (normal lung and advanced LUAD), the cell‐cell communications in between advanced LUAD and other lung samples (normal lung and early LUAD)

Using CellTalker, we identified 4, 20, and 50 unique interactions in normal lung, early LUAD, and advanced LUAD tissues, respectively. In contrast with early LUAD, the pairs GNAI2‐DRD2, GNAI2‐LHCGR, and C4B‐CD46 were only detected in advanced LUAD, while COL3A1‐MAG, FN1‐MAG, HLA‐C‐SLC9C2, and COL2A1‐MAG were uniquely expressed in early LUAD (Figure S9). Our study revealed that compared with normal lung and early LUAD tissues, advanced LUAD had a more unique set of circumstances for cell‐cell communication, which may be a source of the distinctive features of advanced LUAD. The detailed cell‐cell communication pairs are shown in Figure 8 and Table S5.

## 3 DISCUSSION

LUAD, which is characterized as one of the most common and fatal carcinomas in adults, results in enormous economic and medical burdens every year, especially for advanced LUAD.^2^, ^32^ Although great progress in recent decades has been made in LUAD treatments, greatly improving the prognosis for early‐stage LUAD, the overall outcomes for advanced stage LUAD remain poor.^33^, ^34^ Thus, comprehensive investigation of the cell lineages within LUAD lesions is worthwhile.

Here, using single‐cell transcriptome profiles, we included 204,157 cells from normal lung, early LUAD, and advanced LUAD tissues to explore the cellular dysregulation and biological changes in the TME of each LUAD lesion. We constructed a single‐cell atlas for different sample conditions and identified gene expression signatures for each lung tissue condition. We also analyzed alveolar, early‐stage malignant, and advanced‐stage malignant cells across different conditions. Next, we used Bhattacharyya distance to quantitatively compare the similarity of these immune lineages between early and advanced LUAD. CD8+/CTL T cells, regulatory T cells, and follicular B cells, which showed apparent differences between early and advanced LUAD, were also analyzed to characterize shifts in the TME from early to advanced LUAD. Cell‐cell interaction analysis was also applied in our study for potentially applicable in clinical practice for advanced LUAD.

The TME is an important component of tumor tissues; it plays an active role in cancer progression and therapeutic responses.^5^ Our study revealed large differences in CD8+/CTL T cells, regulatory T cells, and follicular B cells between early and advanced LUAD. Strikingly, differential expression analysis showed that CXCL13 was mainly expressed in advanced CD8+/CTL and regulatory T cells, consistent with previous studies.^35^, ^36^ The chemotactic cytokine CXCL13 has been shown to play a critical role in cell proliferation, invasion, and survival.^37^ Within the TME, CXCL13 induces its receptor CXCR5.^38^, ^39^ Biswas et al demonstrated that co‐expression of the ligand‐receptor pair CXCL13–CXCR5 directly regulates the epithelial to mesenchymal transition in tumors and enhances tumor migration and invasion,^40^ indicating that CXCL13 may be a valuable prognostic indicator and a therapeutic target for patients with advanced LUAD.

Heterogeneity is a general characteristic for most carcinomas, especially LUAD.^41^, ^42^ Previous analyses have demonstrated that genomic and transcriptomic alterations are a universal source for ITH in many tumors.^43^ Ma et al^44^ revealed great heterogeneity in LUAD tumor cells and demonstrated that the heterogeneity of immune module expression (IFN‐γ signaling pathway genes) will improve prognoses of immunotherapies. Our study estimated tumor cell‐specific transcriptomic diversity scores and performed GSVA and inferCNV analyses to provide an intuitionistic way to measure intra‐ and inter‐tumor heterogeneity at different stages of LUAD. Since current technologies for whole‐genome or whole‐exome sequencing at the single‐cell level are still not mature enough to apply in reality, CNVs inferred by transcriptome profiles have been extensively applied to evaluate genomic shifts.^45^ In the present study, we found that compared with non‐malignant cells, malignant cells had higher CNV levels. We also noticed that in contrast with early LUAD cells, malignant cells in advanced LUAD had the highest CNV levels, which suggested that advanced LUAD might have a higher level of heterogeneity. Based on scRNA‐seq data, similar results were also found in transcriptional noise and heterogeneity score analyses. Ma et al^41^ showed that patients with tumors with higher heterogeneity levels also had poorer prognoses, and hypoxia‐dependent vascular endothelial growth factor (VEGF) may be a key factor associated with heterogeneity of TME polarization. Previous studies have suggested that hypoxia could induce HIF1A to activate hypoxia signaling, stimulate polarization of fibroblasts, macrophages, and endothelial cells within the TME, and accelerate tumor progression via VEGF.^46^, ^47^ The role of VEGF in heterogeneity has not yet been fully investigated; further studies are needed.

Metabolic reprogramming is an indispensable component of biochemical reactions as it supplies tumor cells' energy to survive and maintain cellular function.^48^ Tumor cells consume a large amount of glucose via glycolysis to obtain energy even in the presence of adequate oxygen; consequently, a large amount of lactic acid produced via the glycolytic pathway promotes an acidic TME.^49^ Previous studies have also revealed that the anti‐tumor immunological effect, which is mainly mediated by tumor‐infiltrating lymphocytes, is weakened within the acidic TME.^50^ In the present study, we found that glycolytic and oxidative phosphorylation pathways were upregulated in early and advanced LUAD cells. Interestingly, compared with early LUAD, we also observed that more cell types were involved in the upregulated glycolytic and oxidative phosphorylation pathways, which may further downregulate anti‐tumor effects in advanced LUAD.

KRAS mutation is thought to be the most common activating lesion in human tumors, especially pancreas, lung, and colon carcinomas, and is considered a predictor of poor patient outcomes.^51^, ^52^ Intriguingly, we found that compared with cells in early LUAD, cells in advanced LUAD were generally enriched in the KRAS_SIGNALING_DN pathway; previous studies have revealed that the multiple genes regulated by KRAS activation play important biological roles in proliferation, metabolism, and TME reprogramming of tumors.^53^ Although there has not yet been successful clinical application of agents targeting KRAS activation, recent studies have suggested drugs that target molecules downstream of KRAS signaling, such as MEK, as novel therapeutic targets for the treatment of KRAS‐activated carcinomas.^54^ Our study provides further insight into KRAS activation at the single‐cell level in advanced LUAD.

There are some limitations to our study. First, we mainly focused on immune cells, which had obvious differences and included more than 400 cells in early and advanced LUAD samples. Second, unlike other studies,^30^, ^55^ the patients included in our study were surgically resected lung cancer patients rather than patients with recurrence and metastasis. However, our research improves our understanding of the TME and cellular heterogeneity in patients with different conditions of LUAD, which will serve as a basic framework and valuable resource for investigating the pathogenesis of LUAD and identifying potential therapeutic targets in the future.

## 4 MATERIALS AND METHODS

### 4.1 Ethics statement

This study was approved by the Ethics Committee of Zhongshan Hospital, Fudan University, China (B2018–137R). Informed consent was obtained when patients were hospitalized.

### 4.2 Study cohorts

A total of 14 primary LUAD patients who received curative surgical resection in the Department of Thoracic Surgery in Zhongshan Hospital (FDZSH) were included for scRNA‐seq analyses. We also integrated the other two independent LUAD patient cohorts, which were downloaded from ArraryExpress (accession numbers E‐MTAB‐6149 and E‐MTAB‐6653) and Human Cell Atlas Data Coordination Platform (accession number PRJEB31843), used in our study. For downstream analysis, LUAD patients were divided into early LUAD (Stage I) and advanced LUAD (Stage II–III).

In bulk RNA analysis, traditional LUAD RNA‐seq data were obtained from six GEO databases (GSE30219, GSE31210, GES3141, GSE37745, GSE50081, and GSE68465). We also selected LUAD patients from TCGA database for bulk RNA analysis.

### 4.3 Tissue processing and single‐cell sequencing

We collected the samples after surgical resection and dissociated the tissues into a single‐cell suspension as described in the Supplement Methods.

### 4.4 10x scRNA‐seq data analysis

Seurat R package^56^ was applied in our study to convert the scRNA‐seq data as a Seurat object. Cells that expressed fewer than 200 genes or more than 7000 genes, or had more than 20% mitochondrial genes, were removed at the quality control step. Data were then normalized by the “NormalizeData” function with the “LogNormalize” method, and “FindVariableFeatures” was used to identify the top 2000 highly variable genes. Next, we used the “RunPCA” function to reduce the dimension of the scRNA‐seq data after scaling and centering features with the “ScaleData” function. The “RunHarmony” function in the “Harmony” R package, which can simultaneously account for multiple factors, was used with default parameters to integrate the different study cohorts in our study.^57^ Subsequently, we used the “RUNUMAP” function to conduct uniform manifold approximation and projection (UMAP) analysis. We also used the “FindClusters” and “FindAllMarkers” functions to conduct cell clustering analysis and detect gene expression markers. Afterwards, we used the SingleR package, CellMarker dataset, and previous studies^11^, ^58^ to annotate the cell types in our study.

The “SubsetData” function was also applied to extract the sub‐cluster for downstream analysis. After detecting clusters and gene expression markers with the “FindClusters” and “FindAllMarkers” functions in sub‐clusters, the “RUNUMAP” function was also used to perform UMAP analysis. The sub‐clusters were annotated as described above.

### 4.5 Construction of a single‐cell transcriptome network

To better investigate the dynamic changes of the overall molecular hallmarks in different stages (normal lung tissues vs early‐stage LUAD vs advanced stage LUAD), a single‐cell transcriptome network was constructed. Briefly, we first identified the centroids, which constitute the most significant number of cells in each network and then assessed the association between centroids and other cell types by Pearson correlation coefficient. Subsequently, we identified the marker genes for each stage with the “FindMarkers” function with a threshold of log2 fold‐change > 0.5 and FDR < 0.05. PPI and functional enrichment analyses were also performed to reveal the molecular and functional changes in different stages.

### 4.6 Construction of a cell to cell interaction network

The R package “CellTalker” is a widely used algorithm. Based on a recently described list of receptors and ligand pairs, “CellTalker” provides a way to construct a cell to cell interaction network. Cell communication analyses were performed as follows: (1) receptor/ligand genes detected in more than 10 cells in each cell type from >50% of patients within a group were selected for downstream analysis and (2) the interactions were evaluated and visualized by the “unique_interactions” and “circos_plot” functions, respectively.

## CONFLICT OF INTEREST

The authors have no conflict of interest to declare.

## AUTHOR CONTRIBUTIONS

Cheng Zhan and Junjie Xi conceived the study. Zhencong Chen, Yiwei Huang, and Zhengyang Hu performed most of the bioinformatics analysis and wrote the manuscript. Mengnan Zhao, Tao Lu Yuansheng Zheng, Jiaqi Liang, Songtao Xu, and Ming Li collected the tumor samples and analyzed the data. Guoshu Bi, Zhengyang Hu, Wei Jiang, and Qun Wang helped project design and manuscript editing. Lijie Tan supervised this study.

## Supporting information

Supporting Information

Supporting Information

Supporting Information

Supporting Information

Supporting Information

Supporting Information

Supporting Information

**Figure S1 Integration of single‐cell data with Harmony**. (A) Cells group by dataset before integration. (B) Harmony plot of cells. (C) After Harmony integration, datasets are mixed together.

**Figure S2 Heatmap showing markers of each cell type and the UMAP plot of alveolar and cancer cells**. (A) The relative expression level of genes across cells is shown, sorted by cell type. (B) UMAP plot of the alveolar and cancer cells profiled here, with each cell color coded for (left to right): its sample type of origin (normal samples or tumor samples), the cell type, and the malignancy scores.

**Figure S3 scPred Analysis to Validate Cell Annotations in Seurat Object**. (A) Probabilities for each cell type versus other cell labels in the trained model. Each panel represents a prediction model and the colors of the known true classes. All other cells are cells except the positive class (for example, for the cancer cells prediction model, all other cells are alveolar and epithelial cells). (B) Distribution of posterior probabilities for cells to belong to the normal class or be unassigned in the prediction model. Each panel represents the predictions each.

**Figure S4 The most enriched pathways for marker genes in other cell types**.

**Figure S5 Functional enrichment analysis for the overall molecular hallmarks implicated for early LUAD and advanced LUAD. Colored by cluster ID or *p*‐value**. i. early LUAD. ii. advanced LUAD.

**Figure S6 Flow cytometry and qRT‐PCR for cancer cells and alveolar cells**. (A) Heatmap showing ROS and apoptotic gene expression signature between advanced and early LUAD groups. (B) Identified and sorted the cancer cells and alveolar cells in the tumor sample and normal sample by flow cytometry. (C) i. The gene expression levels of MPO (*p* < 0.01), FEZ1(*p* < 0.01), PPP2R4(*p* < 0.01), and CREBBP (*p* < 0.01) were significantly increased in advanced LUAD tumor cell. ii. The gene expression levels of TNFSF10 (*p* < 0.01), ECM1 (*p* < 0.01), and RNF213 (*p* < 0.01) were significantly increased in advanced LUAD tumor cells, whereas the expression levels of SCGB3A2 (*p* < 0.01), SCGB3A1 (*p* < 0.01), and SFTPC (*p* < 0.01) were increased in early LUAD.

**Figure S7 Lasso (Least Absolute Shrinkage and Selector Operation) algorithms were performed to select advanced LUAD‐related prognostic genes**. (A) LASSO coefficient profiles of gene markers for advanced LUAD. (B) Partial likelihood deviance is revealed by the LASSO regression model. The vertical dotted lines were drawn at the optimal values by using the minimum criteria and 1‐SE criteria.

**Figure S8 Heatmap showing the expressions of HLA genes among multiple cell types in normal lung tissues and different conditions of LUAD**.

**Figure S9 qRT‐PCR for unique cell‐cell communication pairs in early LUAD and advanced LUAD**. The gene expression levels of FN1 (*p* < 0.01) and MAG (*p* < 0.01) were significantly increased in early LUAD, whereas the expression levels of GNAI2 (*p* < 0.01) and DRD2 (*p* < 0.01) were increased in advanced LUAD.

## Data Availability

The data used in this study can be obtained by request to Cheng Zhan (czhan10@fudan.edu.cn).
