# Supplementary material for: Landscape and dynamics of single tumor and immune cells in early and advanced‐stage lung adenocarcinoma
Source: Clin Transl Med. 2021 Mar 9;11(3):e350. doi: 10.1002/ctm2.350 (PMC7943914; doi:10.1002/ctm2.350)
Supplement: Supplementary file 1 — Supporting Information [file CTM2-11-e350-s009.docx]

**Landscape and dynamics of single tumor and immune cells in early and advanced-stage lung adenocarcinoma**

Zhencong Chen^1#^, Yiwei Huang ^1#^, Zhengyang Hu^1#^, Mengnan Zhao ^1^, Ming Li^1^, Guoshu Bi^1^,

Yuansheng Zheng^1^, Jiaqi Liang^1^, Tao Lu^1^, Wei Jiang^1^, Songtao Xu^1^, Cheng Zhan^1*^, Junjie Xi^1*^,

Qun Wang^1^, Lijie Tan^1^

^1^ Department of Thoracic Surgery, Zhongshan Hospital, Fudan University, No. 180, Fenglin Road, Shanghai, 200032, China

^#^These authors contributed equally: Zhencong Chen, Yiwei Huang, and Zhengyang Hu.

^*^The authors are all corresponding authors of this paper.

**Corresponding authors’ information**

Cheng Zhan and Junjie Xi

Department of Thoracic Surgery,

Zhongshan Hospital, Fudan University,

No. 180, Fenglin Road, Shanghai, 200032, China;

**Phone:** 86-21-64041990; Fax: 86-21-64041990;

**Email:** czhan10@fudan.edu.cn;

xi.junjie@zs-hospital.sh.cn

Funding information

This work was supported by the Research Foundation of Shanghai Municipal Health Commission (20204Y0228), the Science and Technology Fund of Xiamen City(3502Z20184004), and Research Development Fund of Zhongshan Hospital, Fudan University (2019ZSFZ002 and 2019ZSFZ19).

**Supplement Methods**

**Tissue processing**

Following surgical resection, samples were collected for immediate preparation of single-cell suspensions. According to the manufacturer's protocol, to dissociate the tissues into a single-cell suspension, the chopped tissues were digested with a Tumor Dissociation Kit (Miltenyi Biotec, Gladbach, Germany) in MACS C Tubes (130-094-392, Miltenyi Biotec). Then, 40 μm filters were adopted to remove large substances. We then used Red Blood Cell Lysis Solution (10×) (Sigma-Aldrich, St. Louis, MO, USA) and Dead Cell Removal Kit (Miltenyi Biotec) for 10 min with PBS to remove erythrocytes and dead cells, respectively.

**Single-cell sequencing and preprocessing**

Following the manufacturer’s instructions, Chromium Single Cell 3' Reagent kit (version 2) (10×Genomics, Pleasanton, CA, USA) and Chromium Single Cell Controller Instrument (10×Genomics) were adopted for preparation of single-cell RNA-seq libraries. The reverse transcription reactions were conducted to barcode full-length cDNA, and polymerase chain reaction (PCR) with appropriate cycles was conducted to amplify the cDNA. Then, DynaBeadsMyone Silane Beads (Thermo Fisher Scientific, Waltham, MA, USA) were applied to clean up the cDNA. Subsequently, the amplified cDNA was fragmented, end-repaired, A-tailed, index adaptor-ligated, and library amplified. We used the Illumina sequencing platform (HiSeq X Ten; Illumina, San Diego, CA, USA) to sequence the libraries, and 150 bp paired-end reads were generated.

Additionally, to obtain a matrix with the unique molecular identifier (UMI) counts per gene and associated cell barcode, Cell Ranger v 3.0 (10x Genomics) and GRCh38 were applied following single-cell data sequencing.

**Prediction of cell types as malignant cells**

Accurate identification of malignant lung cells is vital in the analysis of sc-RNA data. Thus, we used the R package “scPred”^1^, which is based on variance structure decomposition, to validate our cell identification results. As described in the manual, we first constructed a training dataset that contained half alveolar and cancer cells by the “createDataPartition” function. Then the “eigenDecompose,” “getFeatureSpace,” and “trainModel” functions were applied to complete PCA analysis, obtain cell type-informative principal components, and train the prediction model, respectively. Next, we used the “scPredict” and “getPredictions” functions to predict the cell types of all the remaining cells. Finally, we compared the results in the ‘Seurat object’ and ‘scPred object’ to check the accuracy of cell annotation.

**Heterogeneity score**

To further investigate the level of intra-tumoral heterogeneity, we used a method based on the transcriptome data of malignant cells within the tumor^2^. We first embedded the malignant cells in a new PCA space and included 50 PCs to calculate the heterogeneity score, which was determined as follows:

$$Heterogeneity Score=\frac{1}{m}\sum_{i=1}^{m} \sqrt{\sum_{j=1}^{m} (x_{ij}-\mu_{j})2}$$

Cells with the first three features (i.e., PCs) beyond the range of [$\mu_{j}$-3$\sigma_{j}$, $\mu_{j}$+3$\sigma_{j}$] were removed as the extreme values on diversity score. In our study, $x_{in}$ represents the *i*th malignant cell, $\mu_{j}$ represents the centroid of malignant cells; and $\mu_{j}$ and $\sigma_{j}$ represent the mean and standard deviation of the *j*th feature of cancer, respectively.

**Transcriptional noise**

According to previous studies, transcriptional noise analysis was performed as previously described^3,4^. Only cell types with more than 400 cells in both early or advanced stage LUAD were included. Then, we divided genes into ten equally sized bins and selected genes that had the lowest coefficient of variation in each bin (outliers had been removed). Subsequently, the Euclidean distance, which was used as the index for transcriptional noise, between each cell within each group was evaluated by the “distancevector” function in the “hopach” package. Next, the average value of the Euclidean distances for each patient and transcriptional noise ratio between early or advanced stage LUAD was calculated. Wilcoxon’s rank-sum test was used to statistically compute the relationship between transcriptional noise and lesion stage within each cell type. R function “p.adjust()” was used with the Bonferroni–Hochberg method for p-value correction and adjustment. P-values less than 0.05 were considered significant.

**Estimation of copy number variations**

The estimation of copy number variations (CNVs) in each region was conducted by R package “inferCNV”^5^. We prepared a raw count matrix of single-cell RNA-Seq expression, an annotations file, and a gene/chromosome positions file as the input file. Then, the “CreateInfercnvObject” and “run” functions were applied with default parameters to compute the CNV level. To calculate the malignancy score, R package “scCancer”^6^ and the “getMalignScore” function was applied to calculate the malignancy score.

**Regulome analysis**

To explore the gene regulatory networks in each cell type, R package “SCENIC” was used^7^. We prepared the input file from the Seurat object using the “initializeScenic” function and filtered genes that expressed greater than three UMIs in 1% of cells and were detected in more than 0.1% of cells. The motifs databases (hg19-500bp-upstream-7species.mc9nr.feather and hg19-tss-centered-10kb-7species.mc9nr.feather) were obtained from <https://resources.aertslab.org/cistarget/databases>. The R packages “AUCell,” “RcisTarget,” and “GENIE3” were also used in this study^8^.

**Trajectory analysis**

To perform trajectory analysis, the R package monocle2^9^ was used. The scRNA-seq data (including all cells in the columns and differentially expressed genes in the rows) were extracted from the Seurat object. Then, “newCellDataSet,” “reduceDimension,” and “orderCells” were applied to understand the tumor‐reprogramming processes in single cells. Additionally, the “root cell” was defined by its source and biological function.

**Gene set variation and functional enrichment analysis**

We used the GSVA package^10^ to perform Gene Set Variation Analysis (GSVA) and downloaded the gene sets of pathways from The Molecular Signatures Database (MSigDB) database (http://software.broadinstitute.org/gsea/msigdb/index.jsp). Metascape^11^ (<http://metascape.Org>) was applied to perform Gene Ontology (GO) and Kyoto Encyclopedia of Genes and Genomes (KEGG) pathway analyses. We set the thresholds for functional enrichment analysis as adjusted P < 0.01 and number of enriched genes > 3.

**Estimation of the scores of M1/M2 polarization and pro-/anti-inflammatory ability**

To calculate the M1/M2 polarization and pro-/anti-inflammatory ability of macrophage cells, GSVA analysis was performed. The gene sets associated with the above functions were downloaded from previous studies^12,13^.

**Exploration of ROS and apoptotic gene expression signatures between advanced and early LUAD groups**

To explore ROS and apoptotic gene expression signatures between advanced and early LUAD groups, gene sets of ROS and apoptotic pathways were downloaded from the MSigDB database. We used the ‘FindMarkers’ function in the ‘Seurat’ package to identify differentially expressed ROS and apoptotic genes between advanced and early LUAD groups.

**Survival statistical analysis**

Kaplan–Meier and log-rank tests were used to conduct survival analyses. To detect candidate genes, the Least Absolute Shrinkage and Selector Operation (LASSO) algorithm^14,15^ was used to find the most useful prognostic markers among the gene markers for advanced LUAD by the R package “glmnet”^16^. We used Lambda.min as the cutoff point and the “predict” function to construct the prognostic model. LUAD patients with complete survival data were divided into two groups (> median risk score or ≤ median risk score). Additionally, clinical outcomes and gene expression profiles from the TCGA database were set as the train data, while data from the GEO database was set as test data. The statistical threshold for significance was an adjusted P-value < 0.05.

**Evaluating differences between immune lineages in early and advanced LUAD samples**

To quantify the similarity of the distributions of immune lineages derived from early and advanced LUAD tissues, we calculated the Bhattacharyya distance^17,18^ between cells that originated from different sources as follows: (1) cell subtypes, which contained more than 400 cells in both early and advanced LUAD samples, were selected for measurement of distance in the first step; (2) based on the genes with highly variable, we embedded the selected cells in a new PCA space and included the top 50 PCs to calculate the Bhattacharyya distance; and (3) we sampled 400 cells from each source 100 times and calculated the Bhattacharyya distance as follows:

$$D_{\mathrm{Bhatta}}=\frac{1}{8}\left( \vec{u_{1}}-\vec{u_{2}} \right)^{T}\Sigma^{-1}\left( \vec{u_{1}}-\vec{u_{2}} \right)+\frac{1}{2}\log_{e} (\frac{|\Sigma|}{\sqrt{|\Sigma_{1}||\Sigma_{2}|}})$$

where $\vec{u_{1}}$ and$\vec{u_{2}}$ are the mean vectors of each distribution, and $\Sigma=(\Sigma_{1}+\Sigma_{2})/2$. Additionally, the Bhattacharyya distance between cells randomly included from the sample group was also computed as background distribution. We also used the one-sided Wilcoxon rank-sum test to assess the statistical significance between immune lineages in early and advanced LUAD samples. Adjusted one-sided P-values < 0.05 were considered significant.

**Validation**

A total of 20 normal, 20 early LUAD, and 20 advanced LUAD samples were selected for flow cytometry and quantitative real-time polymerase chain reaction (qRT-PCR) analyses. RNA was extracted with TRIzol reagent (Invitrogen, Carlsbad, CA, USA) and dissolved in DEPC water. We then used the TruSeq® RNA Sample Preparation Kit v2 (Illumina, San Diego, CA, USA) to convert mRNA into a template molecule library. QuantStudio 5 (Thermo Fisher Scientific) was used for sequencing.

For flow cytometry, we first used a kit (Miltenyi Biotec, Gladbach, Germany) to dissociate tumor and normal tissues into single cells, and resuspended the cells in PBS and placed them in FACS tubes. The cell suspension was centrifuged at 200 g and 4°C for 5 min, and the pellet was resuspended in PBS. This washing step was performed twice. Then, allophycocyanin-conjugated mouse anti-human EPCAM (5 µL/106 cells; cat. no.: 566658, BD Biosciences, San Jose, CA, USA), BV421-conjugated mouse anti-human CD45 (5 µL/106 cells; cat. no.: 304022, BioLegend, San Diego, CA, USA), or PE-conjugated mouse anti-human FOLR1 (10 µL/106 cells; cat. no.: FAB5646P, R&D Systems, Minneapolis, MN, USA) was added and the cells were incubated for 1 h. After incubation, the suspension was centrifuged at 200 g and 4°C for 5 min, and the supernatant was discarded and washed three times with PBS. The pellet was resuspended in 2% paraformaldehyde and incubated at 4°C for 20 min. Cells were analyzed by flow cytometry with a FACSAriaIII (BD Biosciences).

| **Primer name** | **Sequence (5’-3’)** |
| --- | --- |
| TNFSF10-F | TGCGTGCTGATCGTGATCTTC |
| TNFSF10-R | GCTCGTTGGTAAAGTACACGTA |
| ECM1-F | GCTTCACGGCTACAGGACAG |
| ECM1-R | GAGGCTTCGGGATAGGGGT |
| RNF213-F | GCTGCTGTGAAAAACGAGAAG |
| RNF213-R | TCCCATTTTGACTCCCCAAATTC |
| SCGB3A2-F | AAGCTGGTAACTATCTTCCTGCT |
| SCGB3A2-R | AGGGGCACTTTGTTGATGAGG |
| SCGB3A1-F | TCCGCTCGTGCTTTCTTAGTG |
| SCGB3A1-R | GAGCCCTCTATGAGGTGGTTC |
| SFTPC-F | CACCTGAAACGCCTTCTTATCG |
| SFTPC-R | TTTCTGGCTCATGTGGAGACC |
| MAG-F | GGTGTCTGGTACTTCAATAGCC |
| MAG-R | CTCTCGTGGACTACTTGGGTG |
| FN1-F | CGGTGGCTGTCAGTCAAAG |
| FN1-R | AAACCTCGGCTTCCTCCATAA |
| GNAI2-F | TACCGGGCGGTTGTCTACA |
| GNAI2-R | GGGTCGGCAAAGTCGATCTG |
| DRD2-F | CCCCGCCAAACCAGAGAAG |
| DRD2-R | TTTTGCCATTGGGCATGGTCT |
| MPO-F | TGCTGCCCTTTGACAACCTG |
| MPO-R | TGCTCCCGAAGTAAGAGGGT |
| FEZ1-F | CCACTGGTGAGTCTGGATGAA |
| FEZ1-R | CGGAAGAAAAATTCTCAAGCTCG |
| PPP2R4-F | TCTCAGGCATACGCTGACTAC |
| PPP2R4-R | GGAGACTCTGTACTCGAAGGT |
| CREBBP-F | CGGCTCTAGTATCAACCCAGG |
| CREBBP-R | TTTTGTGCTTGCGGATTCAGT |

**Reference:**

1 Alquicira-Hernandez, J., Sathe, A., Ji, H. P., Nguyen, Q. & Powell, J. E. scPred: accurate supervised method for cell-type classification from single-cell RNA-seq data. *Genome biology* **20**, 264, doi:10.1186/s13059-019-1862-5 (2019).

2 Ma, L. *et al.* Tumor Cell Biodiversity Drives Microenvironmental Reprogramming in Liver Cancer. *Cancer cell* **36**, 418-430.e416, doi:10.1016/j.ccell.2019.08.007 (2019).

3 Enge, M. *et al.* Single-Cell Analysis of Human Pancreas Reveals Transcriptional Signatures of Aging and Somatic Mutation Patterns. *Cell* **171**, 321-330.e314, doi:10.1016/j.cell.2017.09.004 (2017).

4 Angelidis, I. *et al.* An atlas of the aging lung mapped by single cell transcriptomics and deep tissue proteomics. *Nature communications* **10**, 963, doi:10.1038/s41467-019-08831-9 (2019).

5 Puram, S. V. *et al.* Single-Cell Transcriptomic Analysis of Primary and Metastatic Tumor Ecosystems in Head and Neck Cancer. *Cell* **171**, 1611-1624.e1624, doi:10.1016/j.cell.2017.10.044 (2017).

6 Guo, W., Wang, D., Wang, S., Shan, Y. & Gu, J. *scCancer: a package for automated processing of single cell RNA-seq data in cancer*. (2019).

7 Aibar, S. *et al.* SCENIC: single-cell regulatory network inference and clustering. *Nature methods* **14**, 1083-1086, doi:10.1038/nmeth.4463 (2017).

8 Luo, T. *et al.* A single-cell map for the transcriptomic signatures of peripheral blood mononuclear cells in end-stage renal disease. *Nephrology, dialysis, transplantation : official publication of the European Dialysis and Transplant Association - European Renal Association*, doi:10.1093/ndt/gfz227 (2019).

9 Qiu, X. *et al.* Reversed graph embedding resolves complex single-cell trajectories. *Nature methods* **14**, 979-982, doi:10.1038/nmeth.4402 (2017).

10 Hanzelmann, S., Castelo, R. & Guinney, J. GSVA: gene set variation analysis for microarray and RNA-seq data. *BMC bioinformatics* **14**, 7, doi:10.1186/1471-2105-14-7 (2013).

11 Zhou, Y. *et al.* Metascape provides a biologist-oriented resource for the analysis of systems-level datasets. *Nature communications* **10**, 1523, doi:10.1038/s41467-019-09234-6 (2019).

12 Sun, Y. *et al.* Single-cell landscape of the ecosystem in early-relapse hepatocellular carcinoma. *Cell*, doi:10.1016/j.cell.2020.11.041 (2020).

13 Azizi, E. *et al.* Single-Cell Map of Diverse Immune Phenotypes in the Breast Tumor Microenvironment. *Cell* **174**, 1293-1308.e1236, doi:10.1016/j.cell.2018.05.060 (2018).

14 Tibshirani, R. J. J. J. o. t. R. S. S. S. B. M. Regression Shrinkage and Selection via the LASSO. **73**, 273-282 (1996).

15 Goeman, J. J. L1 penalized estimation in the Cox proportional hazards model. *Biometrical journal. Biometrische Zeitschrift* **52**, 70-84, doi:10.1002/bimj.200900028 (2010).

16 Friedman, J., Hastie, T. & Tibshirani, R. Regularization Paths for Generalized Linear Models via Coordinate Descent. *Journal of statistical software* **33**, 1-22 (2010).

17 Cillo, A. R. *et al.* Immune Landscape of Viral- and Carcinogen-Driven Head and Neck Cancer. *Immunity* **52**, 183-199.e189, doi:10.1016/j.immuni.2019.11.014 (2020).

18 Mohammadi, A. & Plataniotis, K. N. Improper Complex-Valued Bhattacharyya Distance. *IEEE transactions on neural networks and learning systems* **27**, 1049-1064, doi:10.1109/tnnls.2015.2436064 (2016).

19 Lu, X. *et al.* Immune Signature-Based Subtypes of Cervical Squamous Cell Carcinoma Tightly Associated with Human Papillomavirus Type 16 Expression, Molecular Features, and Clinical Outcome. *Neoplasia (New York, N.Y.)* **21**, 591-601, doi:10.1016/j.neo.2019.04.003 (2019).
