# Supplementary material for: Landscape and dynamics of single tumor and immune cells in early and advanced‐stage lung adenocarcinoma
Source: Clin Transl Med. 2021 Mar 9;11(3):e350. doi: 10.1002/ctm2.350 (PMC7943914; doi:10.1002/ctm2.350)
Supplement: Supplementary file 7 — Supporting Information [file CTM2-11-e350-s005.docx]

**Supplementary Figure 1.** **Integration of single-cell data with Harmony.** (A) Cells group by dataset before integration. (B) Harmony plot of cells. (C) After Harmony integration, datasets are mixed together.

**Supplementary Figure 2.** **Heatmap showing markers of each cell type and the UMAP plot of alveolar and cancer cells.** (A) The relative expression level of genes across cells is shown, sorted by cell type. (B) UMAP plot of the alveolar and cancer cells profiled here, with each cell color coded for (left to right): its sample type of origin (Normal samples or Tumor samples), the cell type, and the malignancy scores.

**Supplementary Figure 3.** **scPred Analysis to Validate Cell Annotations in Seurat Object.** (A) Probabilities for each cell type versus other cell labels in the trained model. Each panel represents a prediction model and the colors of the known true classes. All other cells are cells except the positive class (for example, for the cancer cells prediction model all other cells are alveolar and epithelial cells). (B) Distribution of posterior probabilities for cells to belong to the normal class or be unassigned in the prediction model. Each panel represents the predictions each

**Supplementary Figure 4.** **The most enriched pathways for marker genes in other cell types.**

**Supplementary Figure 5. Functional enrichment analysis for the overall molecular hallmarks implicated for early LUAD, and advanced LUAD. Colored by cluster ID or p-value**. i. early LUAD. ii. advanced LUAD.

**Supplementary Figure 6. Flow cytometry and qRT-PCR for cancer cells and alveolar cells**. (A) Heatmap showing ROS and apoptotic gene expression signature between advanced and early LUAD groups. (B) Identified and sorted the cancer cells and alveolar cells in the tumor sample and normal sample by flow cytometry. (C) i. The gene expression levels of MPO (P < 0.01), FEZ1(P < 0.01), PPP2R4(P < 0.01), and CREBBP (P < 0.01) were significantly increased in advanced LUAD tumor cell. ii. The gene expression levels of TNFSF10 (P < 0.01), ECM1 (P < 0.01), and RNF213 (P < 0.01) were significantly increased in advanced LUAD tumor cells, whereas the expression levels of SCGB3A2 (P < 0.01), SCGB3A1 (P < 0.01), and SFTPC (P < 0.01) were increased in early LUAD.

**Supplementary Figure 7. Lasso (Least Absolute Shrinkage and Selector Operation) algorithms were performed to select advanced LUAD related prognostic genes.** (A) LASSO coefficient profiles of gene markers for advanced LUAD. (B) Partial likelihood deviance is revealed by the LASSO regression model. The vertical dotted lines were drawn at the optimal values by using the minimum criteria and 1-SE criteria.

**Supplementary Figure 8. Heatmap showing the expressions of HLA genes among multiple cell types in normal lung tissues and different conditions of LUAD.**

**Supplementary Figure 9. qRT-PCR for unique cell-cell communication pairs in early LUAD and advanced LUAD.** The gene expression levels of FN1 (P < 0.01) and MAG (P < 0.01) were significantly increased in early LUAD, whereas the expression levels of GNAI2 (P < 0.01) and DRD2 (P < 0.01) were increased in advanced LUAD.
