# Supplementary figures and images for: Landscape and dynamics of single tumor and immune cells in early and advanced‐stage lung adenocarcinoma
Source: Clin Transl Med. 2021 Mar 9;11(3):e350. doi: 10.1002/ctm2.350 (PMC7943914; doi:10.1002/ctm2.350)

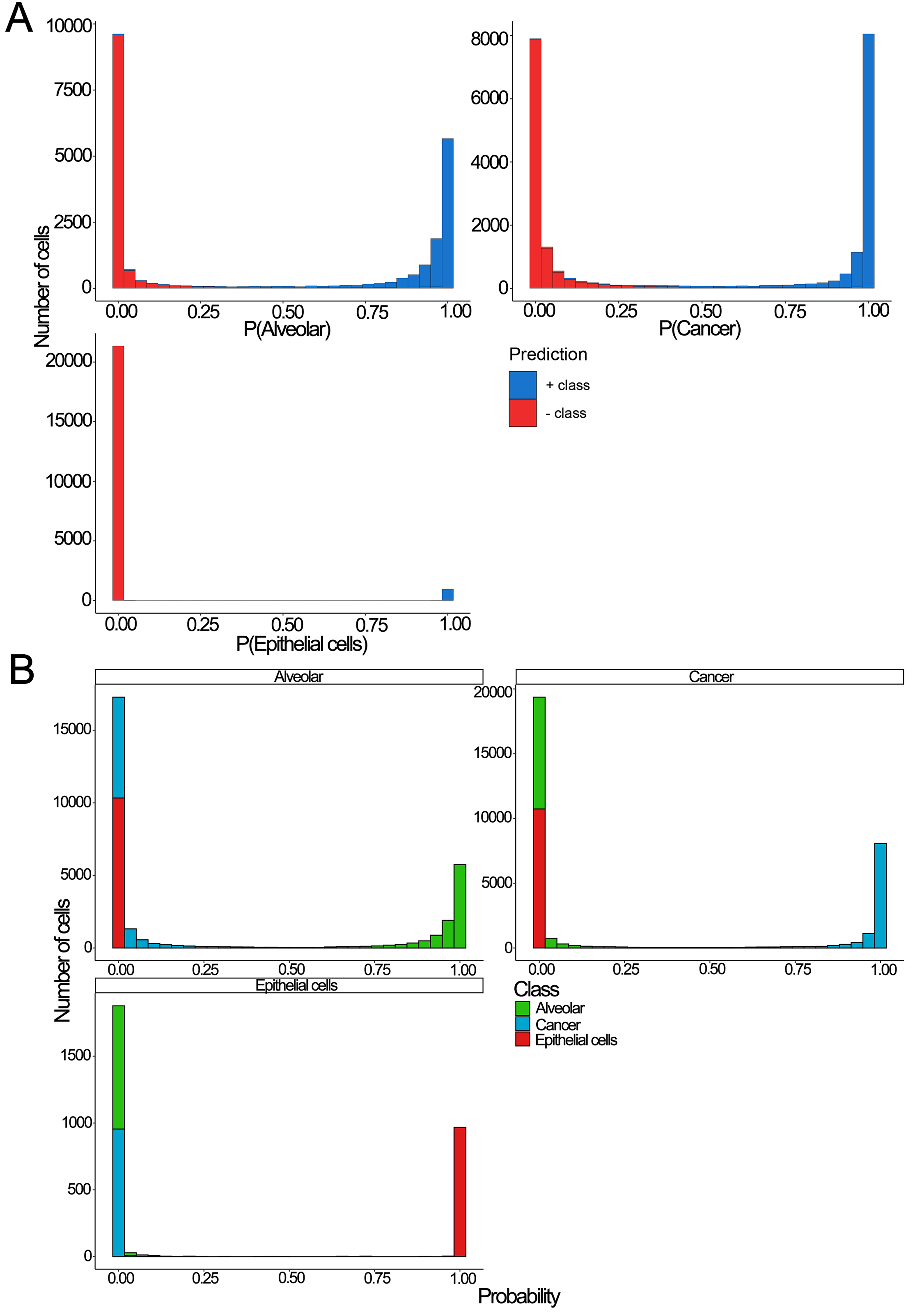

Supplement: Supplementary file 10 — Figure S3 scPred Analysis to Validate Cell Annotations in Seurat Object. (A) Probabilities for each cell type versus other cell labels in the trained model. Each panel represents a prediction model and the colors of the known true classes. All other cells are cells except the positive class (for example, for the cancer cells prediction model, all other cells are alveolar and epithelial cells). (B) Distribution of posterior probabilities for cells to belong to the normal class or be unassigned in the prediction model. Each panel represents the predictions each. [file CTM2-11-e350-s011.tif]

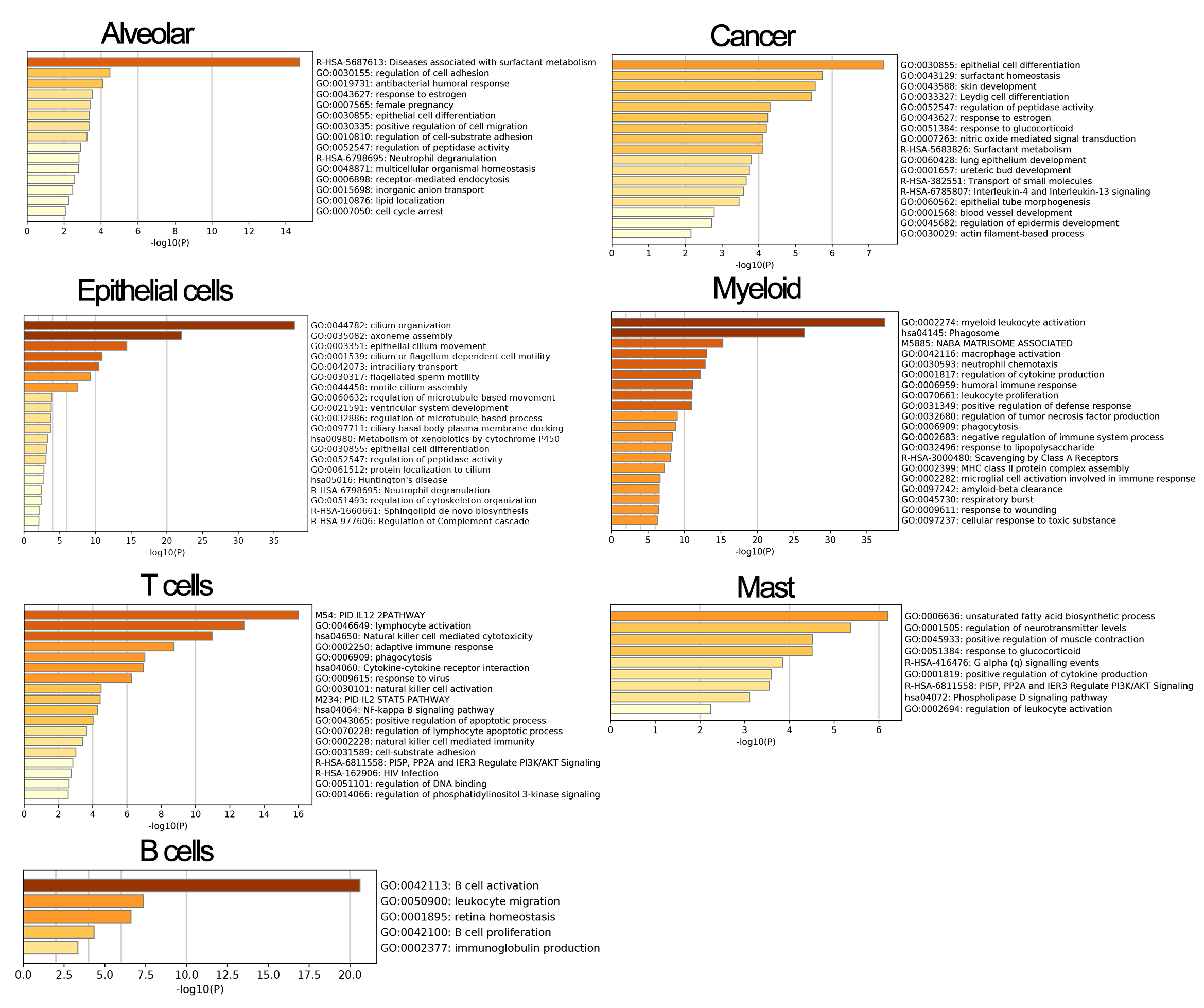

Supplement: Supplementary file 11 — Figure S4 The most enriched pathways for marker genes in other cell types. [file CTM2-11-e350-s015.tif]

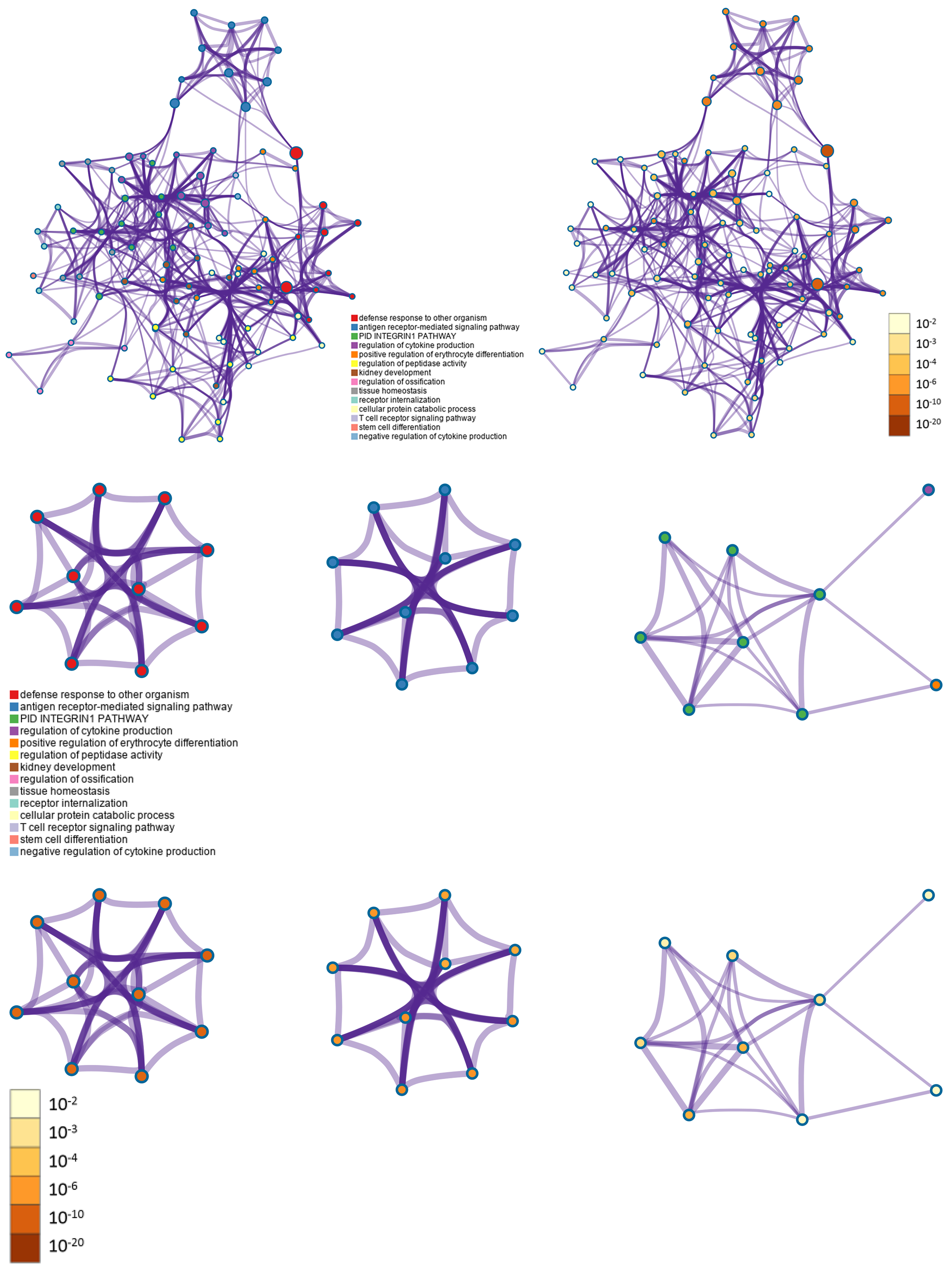

Supplement: Supplementary file 12 — Figure S5 Functional enrichment analysis for the overall molecular hallmarks implicated for early LUAD and advanced LUAD. Colored by cluster ID or p‐value. i. early LUAD. ii. advanced LUAD. [file CTM2-11-e350-s010.tif]

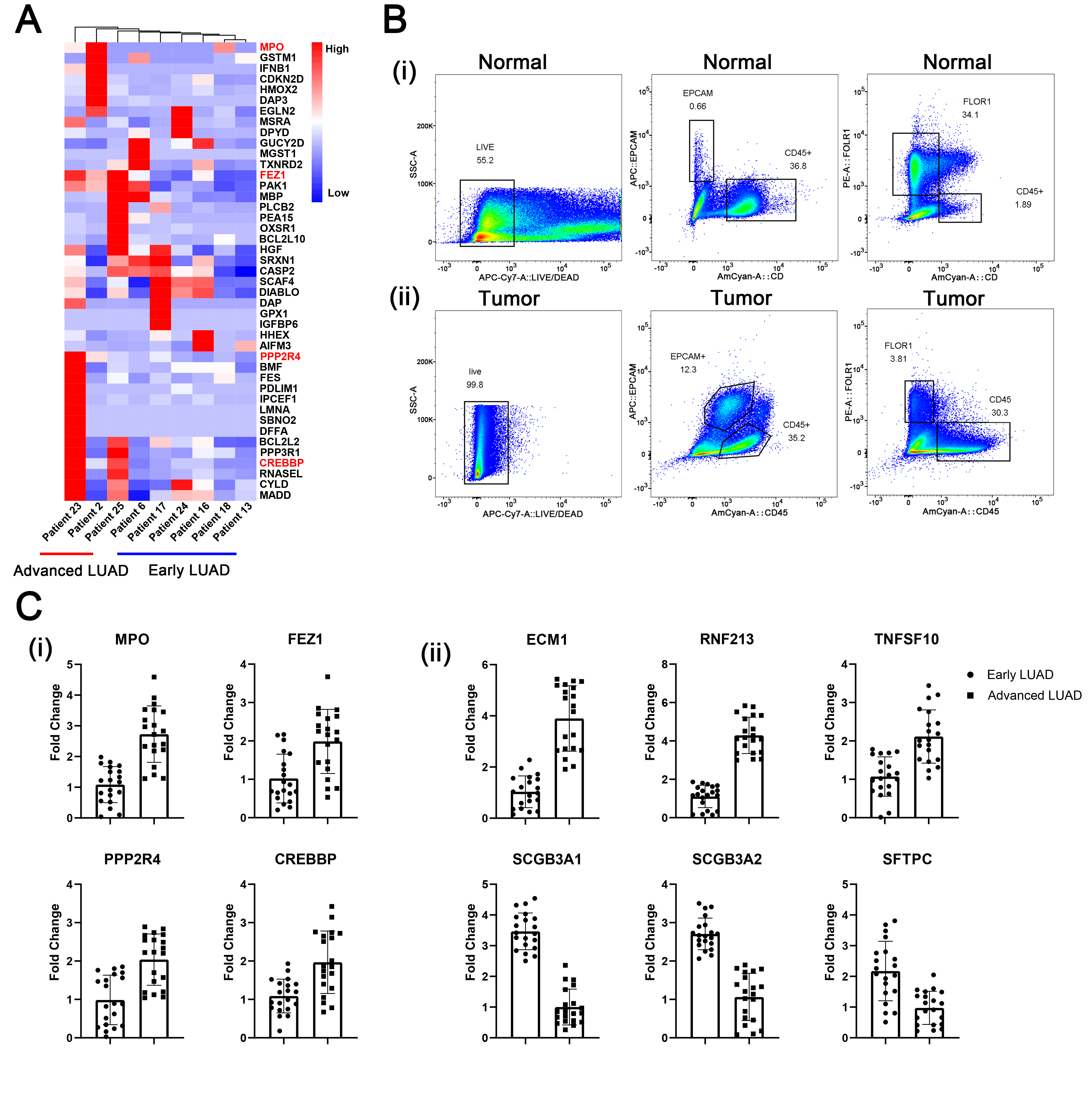

Supplement: Supplementary file 13 — Figure S6 Flow cytometry and qRT‐PCR for cancer cells and alveolar cells. (A) Heatmap showing ROS and apoptotic gene expression signature between advanced and early LUAD groups. (B) Identified and sorted the cancer cells and alveolar cells in the tumor sample and normal sample by flow cytometry. (C) i. The gene expression levels of MPO (p < 0.01), FEZ1(p < 0.01), PPP2R4(p < 0.01), and CREBBP (p < 0.01) were significantly increased in advanced LUAD tumor cell. ii. The gene expression levels of TNFSF10 (p < 0.01), ECM1 (p < 0.01), and RNF213 (p < 0.01) were significantly increased in advanced LUAD tumor cells, whereas the expression levels of SCGB3A2 (p < 0.01), SCGB3A1 (p < 0.01), and SFTPC (p < 0.01) were increased in early LUAD. [file CTM2-11-e350-s008.tif]

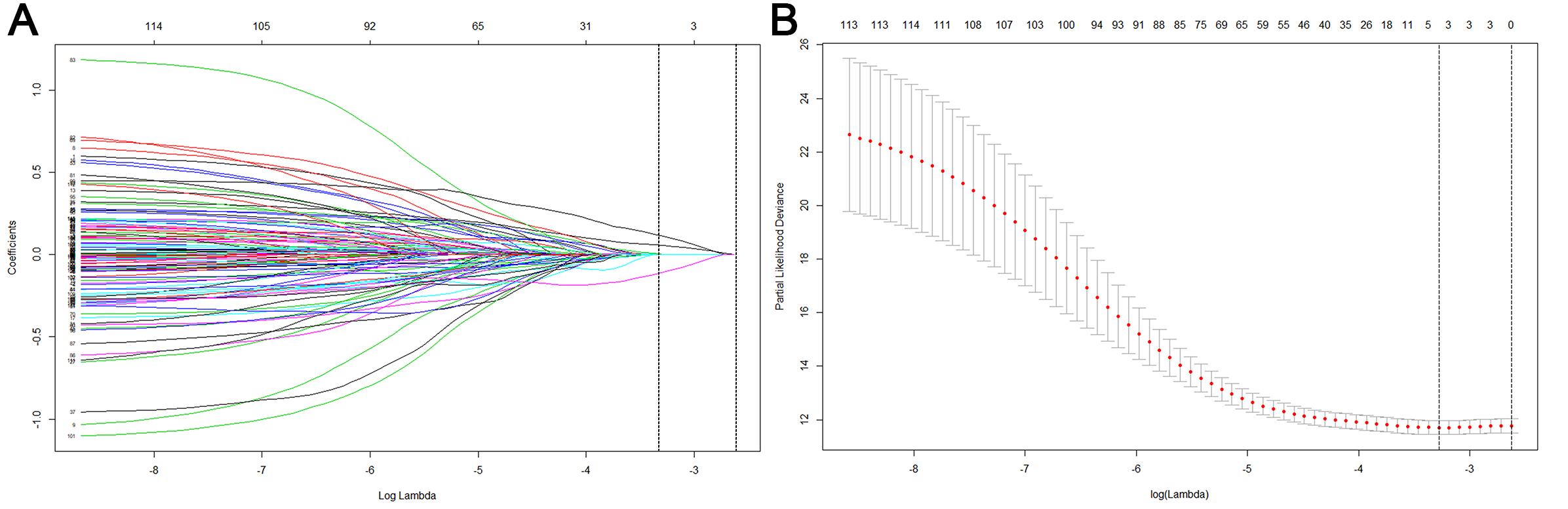

Supplement: Supplementary file 14 — Figure S7 Lasso (Least Absolute Shrinkage and Selector Operation) algorithms were performed to select advanced LUAD‐related prognostic genes. (A) LASSO coefficient profiles of gene markers for advanced LUAD. (B) Partial likelihood deviance is revealed by the LASSO regression model. The vertical dotted lines were drawn at the optimal values by using the minimum criteria and 1‐SE criteria. [file CTM2-11-e350-s001.tif]

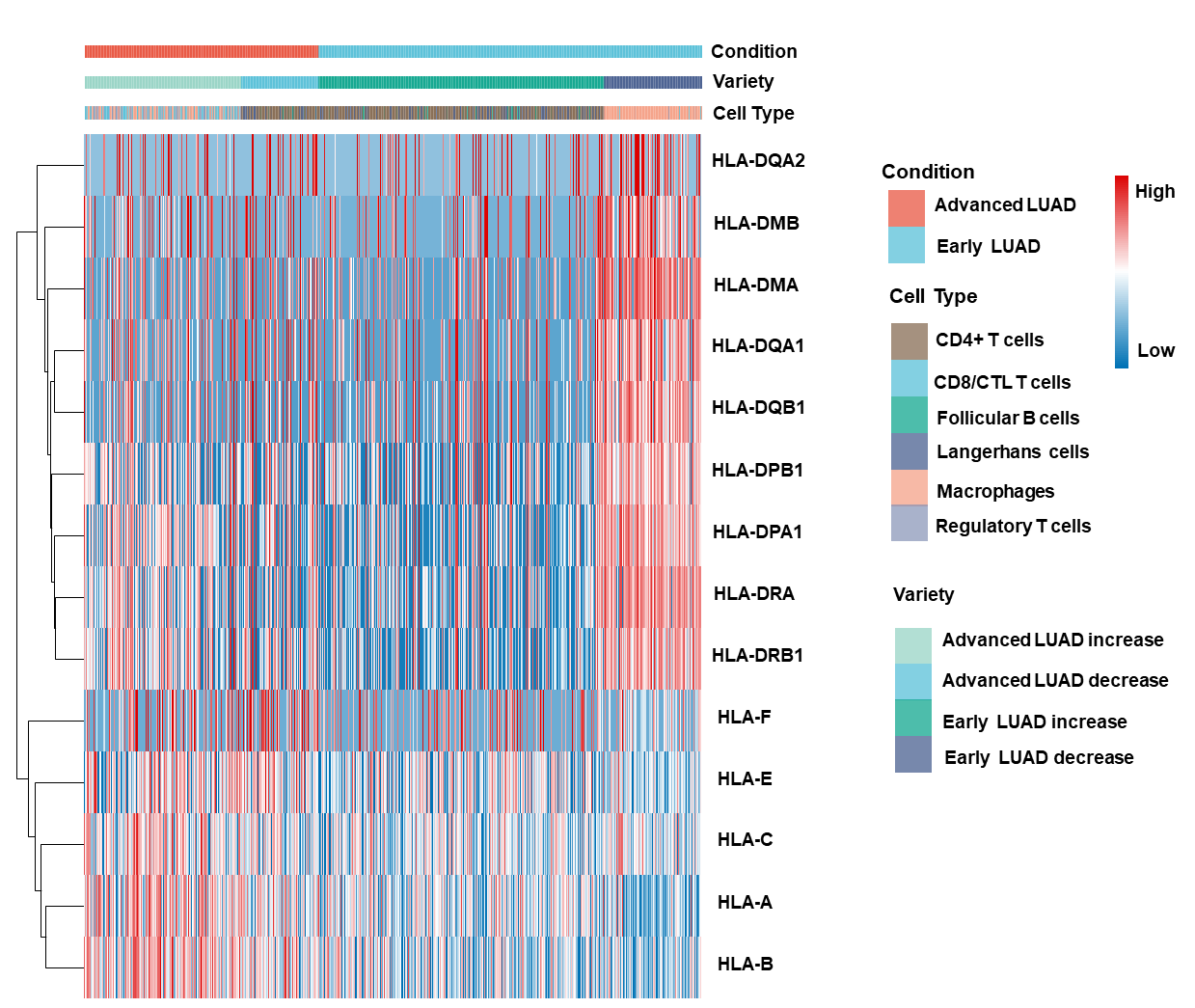

Supplement: Supplementary file 15 — Figure S8 Heatmap showing the expressions of HLA genes among multiple cell types in normal lung tissues and different conditions of LUAD. [file CTM2-11-e350-s003.tif]

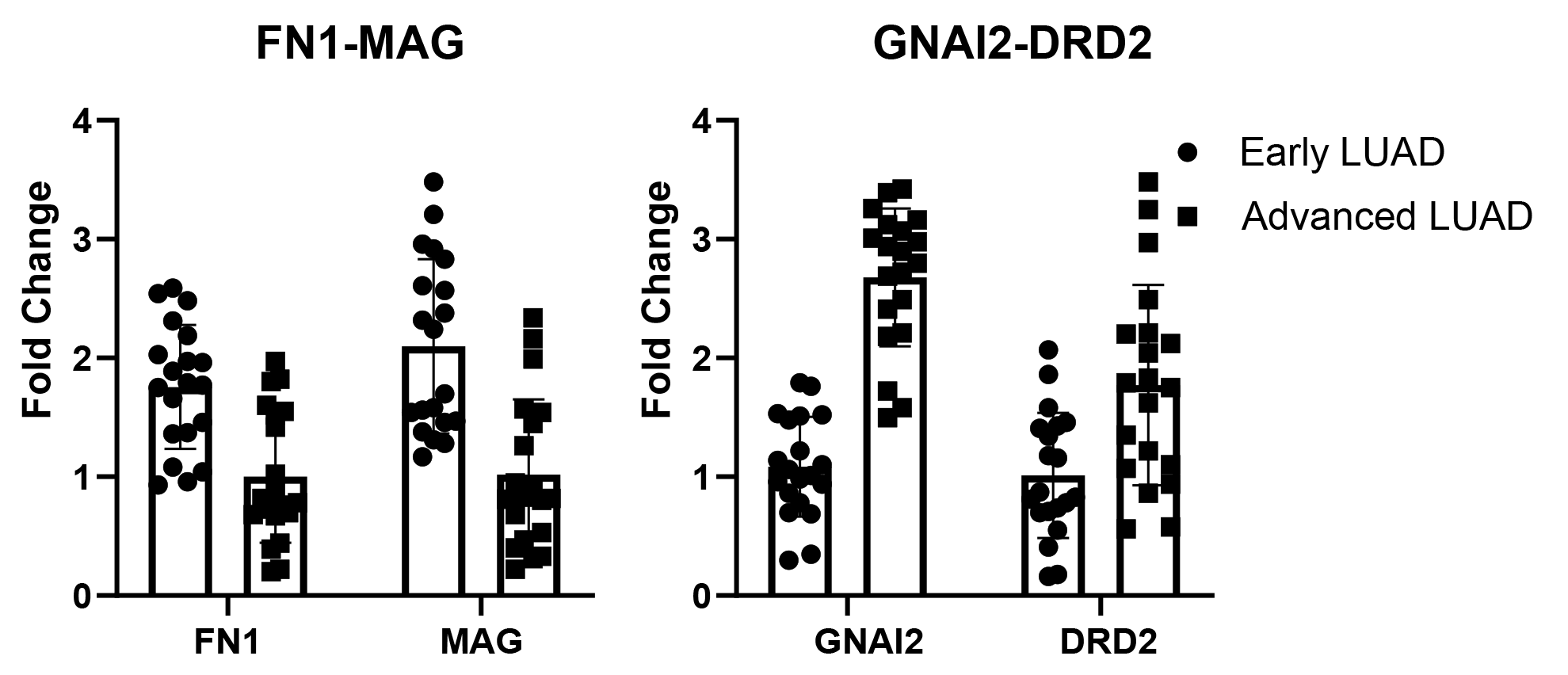

Supplement: Supplementary file 16 — Figure S9 qRT‐PCR for unique cell‐cell communication pairs in early LUAD and advanced LUAD. The gene expression levels of FN1 (p < 0.01) and MAG (p < 0.01) were significantly increased in early LUAD, whereas the expression levels of GNAI2 (p < 0.01) and DRD2 (p < 0.01) were increased in advanced LUAD. [file CTM2-11-e350-s007.tif]
